# Faster but Not Sweeter: A Model of *Escherichia coli* Re-level Lipopolysaccharide for Martini 3 and a Martini 2 Version with Accelerated Kinetics

**DOI:** 10.1021/acs.jctc.4c00374

**Published:** 2024-07-15

**Authors:** Astrid F. Brandner, Dheeraj Prakaash, Alexandre Blanco González, Fergus Waterhouse, Syma Khalid

**Affiliations:** † Department of Biochemistry, University of Oxford, Oxford OX1 3QU, England; ‡ Departamento de Física Aplicada, Universidade de Santiago de Compostela, Santiago de Compostela 15782, Spain; § Departamento de Química Orgánica, Universidade de Santiago de Compostela, Santiago de Compostela 15782, Spain; ∥ MD.USE Innovations S.L., Edificio Emprendia, Santiago de Compostela 15782, Spain

## Abstract

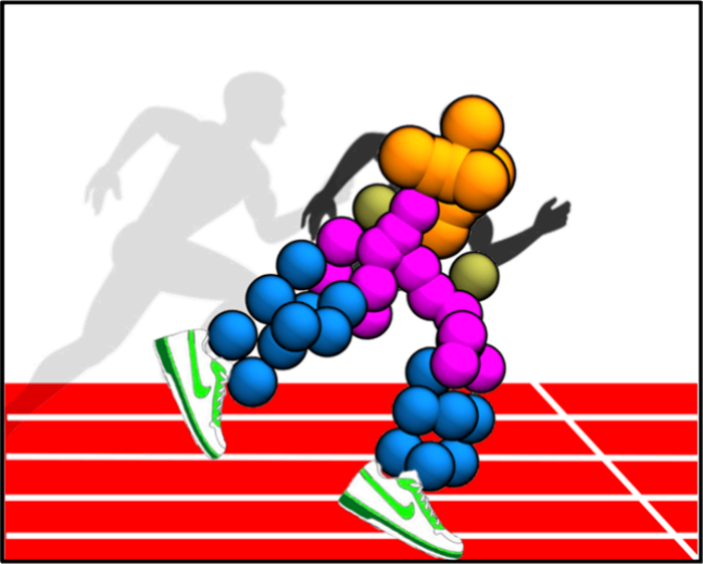

Lipopolysaccharide (LPS) is a complex glycolipid molecule that is the main lipidic component of the outer leaflet of the outer membrane of Gram-negative bacteria. It has very limited lateral motion compared to phospholipids, which are more ubiquitous in biological membranes, including in the inner leaflet of the outer membrane of Gram-negative bacteria. The slow-moving nature of LPS can present a hurdle for molecular dynamics simulations, given that the (pragmatically) accessible timescales to simulations are currently limited to microseconds, during which LPS displays some conformational dynamics but hardly any lateral diffusion. Thus, it is not feasible to observe phenomena such as insertion of molecules, including antibiotics/antimicrobials, directly into the outer membrane from the extracellular side nor to observe LPS dissociating from proteins via molecular dynamics using currently available models at the atomistic and more coarse-grained levels of granularity. Here, we present a model of deep rough LPS compatible with the Martini 2 coarse-grained force field with scaled down nonbonded interactions to enable faster diffusion. We show that the faster-diffusing LPS model is able to reproduce the salient biophysical properties of the standard models, but due to its faster lateral motion, molecules are able to penetrate deeper into membranes containing the faster model. We show that the fast ReLPS model is able to reproduce experimentally determined patterns of interaction with outer membrane proteins while also allowing for LPS to associate and dissociate with proteins within microsecond timescales. We also complete the Martini 3 LPS toolkit for *Escherichia coli* by presenting a (standard) model of deep rough LPS for this force field.

## 1 Introduction

Lipopolysaccharide (LPS) is a major component of the outer membranes of Gram-negative bacteria, and indeed, the outer leaflet of outer membranes contains LPS almost exclusively as the lipidic component.^1^ It is also found in marine-derived aerosols.^2−4^ The complex biochemistry of LPS furnishes it with the ability to form multiple types of noncovalent interactions with other molecules as well as itself. In summary, the acyl tails (there are usually 4 to 6 depending on the bacterial species) can form hydrophobic interactions with similar chemical moieties, whereas the sugars can form multiple hydrogen bonds with polar molecules, and those sugars that are phosphorylated have additional electrostatic interaction capabilities. The multiple interactions neighboring LPS molecules form with each other, the cross-linking of the phosphorylated regions by divalent cations, and the large mass of the molecule all contribute to the limited lateral mobility of LPS.^5^ Indeed, the limited diffusion of LPS is a major contributor to the impermeability of bacterial outer membranes and the difficulty of penetrating these membranes with antimicrobials.^6−8^

Before discussing simulations of LPS, it is useful to describe the molecule in more detail. Full-length LPS is composed of lipid A whose acyl chains face the tails of the inner leaflet phospholipids. Lipid A consists of acyl chains attached to a glucosamine disaccharide, and each of the sugars usually contains a single phosphate group. Covalently linked to lipid A are core oligosaccharides, which in turn are connected to highly diverse O-antigen polysaccharides, and the latter can vary in their number of repeating units (up to ∼100).^9^ ReLPS (also referred to as deep rough) corresponds to lipid A covalently attached to two ketodeoxyoctonic acid (Kdo) sugars, is the minimal viable form of LPS in most Gram-negative bacteria, and thus is often used in experimental and simulation studies.^7,8,10−12^ RaLPS (also referred to as rough LPS) is the name given to the length of LPS that contains all of the core oligosaccharides but not the O-antigen region.^13^ It is important to note that different strains of even the same bacterium can vary in the length of the LPS molecules present in their outer membranes.

Molecular dynamics (MD) simulation studies of LPS can be broadly divided into two categories: those that consider single LPS molecules and those in which LPS-containing bilayers are simulated. The former category includes studies of LPS recognition by host elements such as via the Toll-like receptor (TLR-4) in humans.^14,15^ In such cases, the interaction between a single LPS molecule and the receptor is studied with the simulation initiated with the LPS already bound to the receptor; thus, the encounter between the two molecules is not considered and the slow diffusion of LPS is not a problem. However, it becomes more problematic when multiple LPS molecules are interacting with each other (and other molecules) within a lipid bilayer. Here, the slow lateral motion of LPS necessitates extended equilibration periods compared to simpler phospholipid-containing bilayers. Furthermore, the kinetic barriers to permeation of molecules such as antimicrobial peptides into the LPS-containing bilayers are unsurmountable within the microsecond timescales accessible to MD simulations, despite these peptides being known experimentally to partition into the bilayers.^16^ Coarse-grained (CG) models offer a speed-up compared to all-atom or united-atom models; however, even with the most popular near-atomistic resolution (i.e., not mesoscale) coarse-grained force fields (e.g., Martini^17,18^), breaking beyond the microsecond timescale currently remains impractical. While algorithms for enhanced sampling such as metadynamics and umbrella sampling do offer routes to accessing slower events, we have previously shown that for simulations involving LPS-containing bilayers, this is not straightforward.^5^

Consequently, there is a need for LPS models that exhibit accelerated lateral motion. To advance the currently available coarse-grained toolkit for modeling LPS, here, we present two models of ReLPS: first, a standard model of ReLPS that is compatible with the Martini 3 force field, given that this was omitted from a recently reported Martini 3 parameter set for other levels of LPS,^19^ and second, an accelerated or “fast” ReLPS model that is compatible with the popular Martini 2 coarse-grained suite. Our Martini 3 ReLPS model was developed using our previously reported PyCGTOOL package for the bonded parameters and a manual atomistic-to-coarse-grained mapping procedure, which follows the guidelines suggested by the Martini 3 developers.^20,21^ Models for both force fields are required, given that Martini 2 has the larger repertoire of molecules including lipids and sugars available, whereas Martini 3 is the latest parameter set; however, the range of systems that can be simulated is more limited due to missing parameters for some key molecules (e.g., many lipids and disaccharides). The fast ReLPS model has ∼29 times greater lateral diffusion compared to the standard Martini 2 ReLPS. We show that faster diffusion allows deeper penetration of the antimicrobial polymyxin B1 without rendering the bilayer overly penetrable. Furthermore, we show that our “fast” ReLPS model recapitulates known outer membrane protein–LPS interactions starting from unbiased system configurations, crucially also allowing for protein–LPS dissociation.

## 2 Methods

Atomistic simulations of symmetric ReLPS bilayers were performed with the CHARMM36m force field^22^ to parametrize the new Martini 3 ReLPS. The coarse-grained molecular dynamics (CGMD) simulations presented in this work were performed with the Martini force field version 2.2^17^ or 3.0.^23^ Protein structures used in CGMD simulations were coarse-grained using the martinize.py or martinize2 script for the Martini 2.2 or Martini 3.0 force fields, respectively. An *ad hoc* version of the insane.py script^24^ was used to insert proteins into model membranes. The modifications to the insane.py script include the original ReLPS parameters according to the Martini 2.2 force field as provided by CHARMM-GUI Martini Maker^18^ and our newly parametrized ReLPS (Martini 3) and fast ReLPS (Martini 2). See Table 1 for details on all the simulations presented in this work.

**Table 1:** Details on the Simulation Setup for All Systems Conducted in This Work^a^

**system**	**force field/LPS type**	**production time step (fs)**	**simulated time (μs) [replicas]**	**temp. (K)**	**membrane composition (OL: outer leaflet, IL: inner leaflet); ions**	**embedded proteins or free molecules at tim**e = 0 **(number of molecules)**
symmetric ReLPS bilayer	C36m	2	2.4	320	100% ReLPS; Ca^2+^, K^+^, Cl^–^	
symmetric ReLPS bilayer	M3	10	10 [× 3]	320	100% ReLPS; Ca^2+^, Na^+^, Cl ^–^	
symmetric ReLPS bilayer	M2	10	10 [× 3]	320	100% ReLPS; Ca^2+^, Na^+^, Cl ^–^	
symmetric ReLPS bilayer	M2 fast	10	10 [× 3]	320	100% ReLPS; Na^+^, Cl^–^	
OM + DOPC	M2	20	10 [× 10]	313	OL: 100 ReLPS, IL: 90/5/5 POPE/POPG/CDL; Ca^2+^, Na^+^, Cl^–^	DOPC (1)
OM + DOPC	M2 fast	20	10 [× 10]	313	OL: 100 ReLPS, IL: 90/5/5 POPE/POPG/CDL; Na^+^, Cl^–^	DOPC (1)
OM + DOPC	M3	20	10 [× 10]	313	OL: 100 ReLPS, IL: 90/5/5 POPE/POPG/CDL; Ca^2+^, Na^+^, Cl^–^	DOPC (1)
OM + DPC	M2	20	10 [× 10]	313	OL: 100 ReLPS, IL: 90/5/5 POPE/POPG/CDL; Ca^2+^, Na^+^, Cl^–^	DPC (1)
OM + DPC	M2 fast	20	10 [× 10]	313	OL: 100 ReLPS, IL: 90/5/5 POPE/POPG/CDL; Na^+^, Cl^–^	DPC (1)
OM + DPC	M3	10	10 [× 10]	313	OL: 100 ReLPS, IL: 90/5/5 POPE/POPG/CDL; Ca^2+^, Na^+^, Cl^–^	DPC (1)
OM + octane	M2	20	10 [× 10]	313	OL: 100 ReLPS, IL: 90/5/5 POPE/POPG/CDL; Ca^2+^, Na^+^, Cl^–^	octane (4)
OM + octane	M2 fast	20	10 [× 10]	313	OL: 100 ReLPS, IL: 90/5/5 POPE/POPG/CDL; Na^+^, Cl^–^	octane (4)
OM + octane	M3	10	10 [× 10]	313	OL: 100 ReLPS, IL: 90/5/5 POPE/POPG/CDL; Ca^2+^, Na^+^, Cl^–^	octane (4)
OmpF + 7 LPS	M2	20	12 [× 6]	313	OL: 100 ReLPS, IL: 90/5/5 POPE/POPG/CDL; Ca^2+^, Na^+^, Cl^–^	OmpF trimer (1)
OmpF + 7 LPS	M2 fast	20	12 [× 6]	313	OL: 100 ReLPS, IL: 90/5/5 POPE/POPG/CDL; Na^+^, Cl^–^	OmpF trimer (1)
OmpF + 7 LPS	M3	10	12 [× 6]	313	OL: 98/2 POPE/ReLPS, IL: 90/5/5 POPE/POPG/CDL Ca^2+^, Na^+^, Cl^–^	OmpF trimer (1)
OM + 7x OmpF	M2	20	2 [× 3]	313	OL: 100 ReLPS, IL: 90/5/5 POPE/POPG/CDL Ca^2+^, Na^+^, Cl^–^	OmpF trimer (7)
OM + 7x OmpF	M2 fast	20	2 [× 3]	313	OL: 100 ReLPS, IL: 90/5/5 POPE/POPG/CDL Na^+^, Cl^–^	OmpF trimer (7)
OM + 7x OmpF	M2 standard + fast (50/50%)	20	2 [× 6]	313	OL: 100 ReLPS, IL: 90/5/5 POPE/POPG/CDL; ions: Ca^2+^, Na^+^, Cl^–^	OmpF trimer (7)
OMP island	M2	20	2 [× 3]	313	OL: 100 ReLPS, IL: 90/5/5 POPE/POPG/CDL; Ca^2+^, Na^+^, Cl^–^	OMPs (218)
OMP island	M2 fast	20	2 [× 3]	313	OL: 100 ReLPS, IL: 90/5/5 POPE/POPG/CDL; Na^+^, Cl^–^	OMPs (218)
OMP island	M3	10	2 [× 3]	313	OL: 100 ReLPS, IL: 90/5/5 POPE/POPG/CDL; Ca^2+^, Na^+^, Cl^–^	OMPs (218)
OM + PMB1	M2	10	5 [× 3]	313	OL: 100 ReLPS, IL: 90/5/5 POPE/POPG/CDL; Ca^2+^, Na^+^, Cl^–^	PMB1 (95)
OM + PMB1	M2 fast	10	5 [× 3]	313	OL: 100 ReLPS, IL: 90/5/5 POPE/POPG/CDL; Na^+^, Cl^–^	PMB1 (95)

a The abbreviations are as follows: DPC: dodecylphosphorylcholine; POPE: palmitoyl-2-oleoyl-*sn*-glycero-3-phosphoethanolamine; POPG: 1-palmitoyl-2-oleoyl-*sn*-glycero-3-phosphoglycerol; CDL: cardiolipin; PMB1: polymyxin B1; OM: outer membrane; OMP: outer membrane protein; OL: outer leaflet; IL: inner leaflet. Force field abbreviations are as follows: C36m: CHARMM36m; M2: Martini 2 standard; M2 fast: fast Martini 2; M3: Martini 3.

### 2.1 Atomistic Simulations and Martini 3 Parametrization

Atomistic simulations of a symmetric ReLPS bilayer were set up with CHARMM-GUI^18^ using the CHARMM36m force field. The initial box size was 15 × 15 × 19 nm^3^ containing the membrane (119 ReLPS molecules in each leaflet) solvated in a 0.15 M KCl solution. Seven-hundred fourteen Ca^2+^ ions were included in the ReLPS headgroup and core regions to neutralize charges. The standard protocol of CHARMM-GUI was followed for minimization and equilibration in the NPT ensemble (320 K, 1 bar). A detailed description can be found in Supplementary Methods. The production run was conducted for 2.4 μs.

The Martini 2 fast ReLPS was developed by scaling down the ReLPS–ReLPS Lennard-Jones parameters to reduce the attraction between the headgroup particles (as shown in Figure S11A) such that the area per lipid recorded for the “standard” Martini 2 model of ReLPS was not altered by more than 5% by the scaling. The strength of dispersion/repulsion is defined within the Martini 2 force field as “levels”. Our fast ReLPS model incorporates particle types for the first Kdo sugar attached to lipid A (Figure S11A, B) that are scaled such that they are two levels more repulsive than the particles in the standard Martini 2 ReLPS model. The resultant epsilon parameters are altered by 0.74–0.87x; full details are provided in Figure S11B. The charges on all anionic particles in our fast ReLPS model are scaled down by 50%.

**Figure 1:**
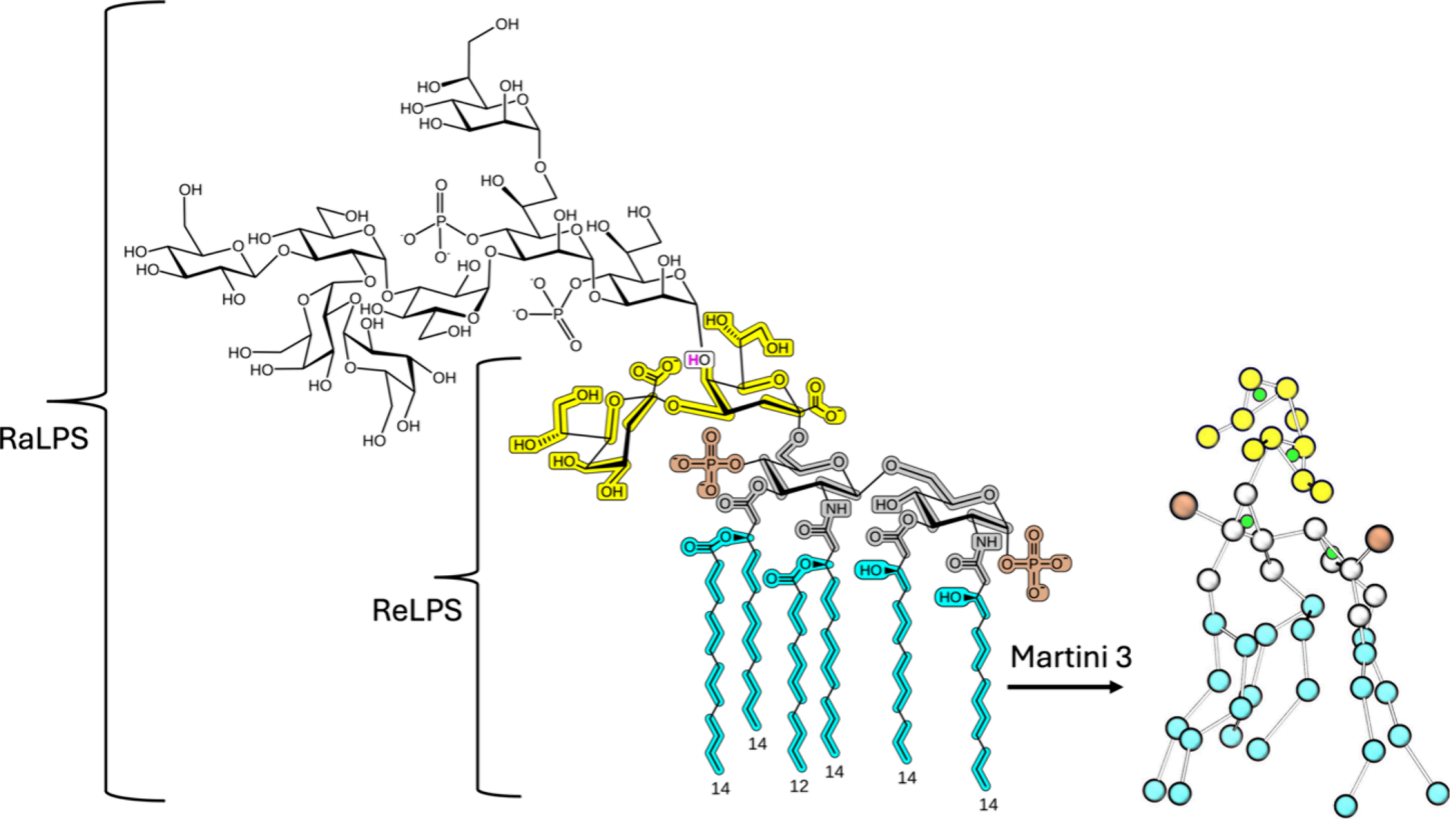
Martini 3 ReLPS mapping. Groups of two to four heavy atoms were mapped into coarse-grained beads. Notably, the sugar rings were modeled with an extra virtual site bead (depicted in green) to better characterize their volume and interactions. Here, each highlighted segment of the 2D depiction corresponds to the same-colored bead on the right CG molecule taken from a simulation snapshot. The four different colors represent the following: ketodeoxyoctonic acid (Kdo) sugars, i.e., first two in core region (yellow), d-glucosamine (GlcN) sugars from the lipid A moiety (gray), phosphate substituents (brown), and acyl chains (cyan). A detailed atom list from all-atom to CG mapping is given in Table S1 and the bead types annotated in Figure S1. The sugars that correspond to RaLPS are added for reference without any associated mapping. The white highlighted box containing a magenta-colored hydrogen atom in one of the Kdo is mapped to Martini 3. That hydrogen would not be present in a RaLPS molecule, as an O-glycosidic bond would be formed instead with the attached heptose molecule.

Coarse-grained coordinates and initial bonded parameters for the Martini 3 ReLPS model were generated from the atomistic simulation following the mapping scheme shown in Figure 1 via PyCGTOOL.^25^ Briefly, first, a mapping scheme from all-atom to coarse grain was chosen to define the CG topology and to map coordinates from a reference atomistic trajectory. Second, coarse-grained bonded parameters (equilibrium distances and force constants) were obtained via the modified Boltzmann inversion technique implemented in PyCGTOOL. Crucially, PyCGTOOL uses distributions of bonded terms from atomistic simulations rather than single values from static structures. A detailed atom list from all-atom to CG mapping and the final parameters are provided in Tables S1–S3 and additional text within the Supporting Information. A time step of 10 fs was chosen a consequence of the resolution of the mapping scheme. In the current version, certain “tiny beads” (i.e., those beads with mass = 36 a.m.u, which represent 2 heavy atoms in Martini 3) are connected via a bond potential and not a constraint, producing a bond with an estimated oscillation period of, e.g., 0.15 ps, which is smaller than 10 times the time step, which should be avoided for energy conservation between time steps. We note here that according to Berendsen and co-workers, a general guideline for the Verlet leapfrog integration scheme is a minimum of five numerical integration steps per period of harmonic oscillation for integration with reasonable accuracy.^26^

Martini 3 parameters for dodecyl phosphocholine (DPC) implemented here are according to those proposed by Cabezudo et al.^27^

### 2.2 Coarse-Grained Simulations

#### 2.2.1 General Simulation Protocols

All CGMD simulation systems were solvated with the (standard) Martini water model before adding 0.15 M NaCl. Charges were neutralized by replacing water beads with either divalent or monovalent cations or both according to the concentration (0/50/100%) of ReLPS and the type of ReLPS (standard/fast Martini 2) used in the membrane. All simulation systems with 100% standard (Martini 2 or 3) ReLPS in the outer leaflet were neutralized with divalent cations (Ca^2+^) in addition to 0.15 M NaCl, whereas 100% fast (Martini 2) ReLPS-containing systems were neutralized with monovalent cations (Na^+^) in addition to 0.15 M NaCl.

##### 2.2.1.1 Martini 2

Details of energy minimization and equilibration are provided in Supplementary Methods. For production simulations, at least 3 replicas were performed, each initiated from the end of the equilibration stage. The simulation protocols for equilibration, details of which are given in Supplementary Methods, were maintained for the production simulations, but for Martini 2 simulations, pressure coupling was achieved via the Parrinello–Rahman barostat^28^ with a time constant of 12 ps.

##### 2.2.1.2 Martini 3

Simulations performed with the Martini 3 force field were equilibrated after minimization as stated in Supplementary Methods. In contrast with simulations with Martini 2, the reaction-field method was used to treat electrostatic interactions. At least 3 replicas were performed for production simulations with a 10 fs time step using the Parrinello–Rahman barostat. They were initiated from the end of the equilibration stage.

#### 2.2.2 *E. coli* Outer Membrane Model Composition

The composition of the outer membrane in CGMD simulations reported here was as follows: palmitoyl-2-oleoyl-*sn*-glycero-3-phosphoethanolamine (POPE)/1-palmitoyl-2-oleoyl-*sn*-glycero-3-phosphoglycerol (POPG)/cardiolipin with a −2 net charge (CDL2): 90/5/5 for the inner leaflet and 100% ReLPS in the outer leaflet. A special set of CGMD simulations contained 2% ReLPS and 98% POPE in the outer leaflet to help accelerate diffusion of lipids to better assess binding and unbinding of ReLPS with outer membrane proteins (OMPs). The X-ray crystal structure of OmpF used in this work was resolved at 1.98 Å (PDB: 3K1B).^29^

#### 2.2.3 Membrane Permeability Tests with Octane, DPC, and DOPC

Simulations were performed to test the spontaneous insertion of either octane, dodecyl phosphorylcholine (DPC), or dioleoyl-*sn*-glycero-3-phosphocholine (DOPC) in standard and fast Martini 2 ReLPS-containing outer membranes. Octane, DPC, or DOPC molecules were initially placed in the solvent phase proximal to the membrane surface but without direct contact with the membrane. Four octane molecules were simulated in each simulation replica, whereas each simulation of DPC or DOPC insertion contained only 1 molecule to avoid potential micelle formation. Position restraints were applied to octane/DPC/DOPC during the equilibration stage. During production, a flat-bottom potential of 1000 kJ/mol/nm was applied to all their beads 5 nm from their equilibrated positions along the *Z*-coordinate to prevent them from inserting into the inner leaflet containing phospholipids. Ten production simulations of 10 μs each were performed for octane, DOPC, and DPC.

#### 2.2.4 PMB1 Simulations with Martini 2

To study the effect of our fast ReLPS parameters on the interactions of polymyxin B1 (PMB1) with the outer membrane, a 20 × 20 nm^2^ membrane composed of 100% ReLPS in the outer leaflet and a mixture of 90% POPE + 5% POPG + 5% cardiolipin in the inner leaflet was constructed using the online CHARMM-GUI Martini Maker.^30^ Charges were neutralized using divalent cations, and 0.15 M NaCl was added to the system. To this membrane, multiple PMB1 were added in random positions and orientations in the aqueous phase above the outer leaflet such that a ratio of 6.6 ReLPS to 1 PMB1 was obtained, following one of the proportions reported in a previous study.^8^ The charges introduced by the peptide were neutralized using Cl^–^ ions.

This system was equilibrated following the standard protocol provided by CHARMM-GUI and was adapted to the presence of PMB1 in the system. Specifically, position restraints were added to the peptides so that they would remain in the solvent and not diffuse toward the membrane during the equilibration steps. Once the system reached a state that allowed simulations to run without numerical errors at an appropriate time step, three starting points were generated for further simulations: The first starting point remained unchanged from the original system, while the second starting point involved the replacement of half of the standard ReLPS molecules with fast ReLPS, ensuring a uniform distribution. Last, the third starting point featured a membrane where all standard ReLPS molecules were substituted with fast ReLPS. As mentioned previously, it is important to note that in simulations where fast ReLPS was introduced, an equivalent amount of divalent cations had to be exchanged for monovalent cations due to differences in ReLPS parametrization. This procedure ensured that the three simulations (3 replicas each) began from identical particle configurations, with the only difference being the presence and quantity of fast ReLPS. Subsequently, these three systems were prepared for a 5 μs simulation without any restraints applied to the system.

#### 2.2.5 Simulations with Multiple OmpFs

We set up simulations containing 7 OmpF trimers arranged in a hexagonal lattice based on atomic force microscopy (AFM) experiments^12^ but placed at approximately 13 nm from each other. They were inserted into a membrane containing 100% ReLPS concentration (with an area per ReLPS lipid initially set to 1.3 nm using the insane.py script) in the outer leaflet. In addition, we performed a set of simulations containing 50% standard and 50% fast ReLPS where one setup contained a uniform distribution of standard and fast ReLPS, and the other had a non-uniform distribution. In these 50% standard/fast ReLPS-containing simulations, a sufficient number of divalent cations had to be replaced with monovalent cations in addition to the 0.15 M NaCl salt concentration due to the requirement of our fast ReLPS model.

Details of analyses and visualization are provided in the Supporting Information. All simulations in this study were performed with the GROMACS simulation suite v2019.4 or higher.^31^ Information on specific GROMACS versions used for production simulations and box dimensions for each system is provided in Table S4.

## 3 Results

### 3.1 ReLPS Model in Martini 3

The current CGMD LPS toolkit has recently been extended by the publication of an *Escherichia coli* LPS parameter set, which is compatible with Martini 3; this includes lipid A and LPS at the Ra- and O-antigen levels^19^ but not ReLPS, which is the minimal level of LPS with which *E. coli* cells are viable. Therefore, we first sought to address this by parametrizing a Martini 3-compatible model of ReLPS. Briefly, a long atomistic simulation of a pure ReLPS membrane patch neutralized with Ca^2+^ ions and in physiological salt concentration was used to obtain the first set of coordinates and bonded parameters based on the manually assigned mapping strategy (Figure 1). The parameters are provided in the Supporting Information as well as the probability distribution functions of each bond, angle, and dihedral present in the Martini 3 model compared to their CHARMM36m counterpart. The detailed process of parametrization is explained in Section 2. In addition, a comparison of the solvent-accessible surface area (SASA) of the Martini 3 vs CHARMM36m models is provided in Figure S5. The SASA computed for the ReLPS molecules in the context of the symmetric bilayer shows good overall agreement with its atomistic counterpart. Despite the Martini 3 SASA distribution being slightly shifted toward larger values, the difference in SASA value between Martini 3 and the atomistic reference model is 0.75 ± 2.6 nm^2^, where the propagated error is larger than the difference, thus showing good agreement between the models.

**Figure 2:**
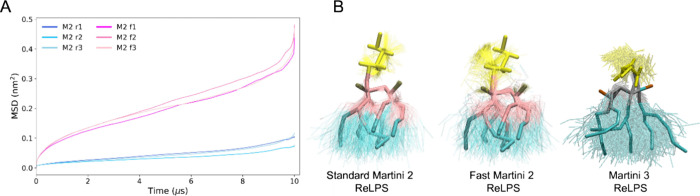
Lateral diffusion and conformational lability of the standard and fast Martini 2 ReLPS models. (A) Comparison between the lateral mean square displacement of individual simulation replicas of standard (M2 r1–r3) and fast Martini 2 (M2 f1–f3) ReLPS models. (B) Structural representations of ReLPS conformational flexibilities for each of the force fields used in this work (overlaying snapshots every 100 ns after fitting in the central part of the ReLPS structure for clarity). See Figure S14A–C for RMSD data on the structural flexibility of ReLPS and Figure S14D for detailed MSD plots. The Martini 3 model follows the same color scheme as in Figure 1.

Comparison of the physical properties of this model with our previously published Martini 2 ReLPS and the reference atomistic (CHARMM36m) versions reveals good agreement and is within the same range of agreement as the aforementioned Martini 3 LPS models. The details of these properties are discussed below in the context of comparison with the subsequently parametrized “fast” Martini 2 ReLPS model (Figure 2).

### 3.2 “Fast” Martini 2 ReLPS Model

Given the limited lateral diffusion of LPS, which is in part due to strong electrostatic interactions mediated by cross-linking of phosphorylated regions by divalent cations, we present our fast ReLPS model compatible with the Martini 2 force field. To parametrize this, we ran coarse-grained (Martini 2) simulations in an outer membrane model containing 100% ReLPS in the outer leaflet and phospholipids in the inner leaflet. Initially, to yield greater lateral diffusion of Martini 2 ReLPS molecules with minimal deviation from canonical physical properties of the outer membrane model, we obtained two ReLPS models where (i) the charges of its anionic beads were scaled down and neutralizing divalent cations were replaced with monovalent cations, and (ii) Lennard-Jones (LJ) potentials (inter-ReLPS) were scaled down (more details provided in Supplementary Methods and associated Figures S6–S11). Next, we combined these models to obtain our “fast” Martini 2 ReLPS model. We summarize the modified bead names and types in Figure S11A and the differences in the LJ potential matrices between standard and fast Martini 2 ReLPS in Figure S11B. This “fast” Martini 2 model of ReLPS was evaluated by computing its mean square displacement (MSD, revealing an ∼29-fold increase along the membrane plane) (Figure S12A), nearest neighbor index (NNI, a measure of lipid mixing revealing an increased value by a factor larger than 4) (Figure S12B), and partial density profiles (Figure S12C), which showed good agreement with those of the standard Martini 2 model.

We also simulated outer membrane models (with 7x OmpF trimers) containing a combination of fast and standard Martini 2 ReLPS molecules (50% each) in the outer leaflet and assessed its effect on area per lipid (APL) (Figure S13A), membrane thickness (Figure S13B), and MSD (Figure S13C). These results were compared to simulations with 100% fast or 100% standard Martini 2 ReLPS. Note that we performed these simulations starting from two distinct initial setups where the fast and standard ReLPS molecules were distributed (i) uniformly or (ii) non-uniformly (Figure S13D). From these simulations, we observed that combining fast and standard ReLPS types starting from either the uniform or non-uniform distribution did not achieve any significant increase in lateral diffusion compared to the standard ReLPS. APL and membrane thickness analyses also revealed that mixing fast and standard ReLPS types tends to provide results comparable to that of pure standard ReLPS. Therefore, from this point onward, we only simulated membranes containing either 100% standard or fast ReLPS.

Comparison of symmetric bilayers of the standard Martini 2 and fast Martini ReLPS revealed stark differences in the lateral mobility and conformational dynamics. The fast Martini 2 ReLPS exhibits about 10-fold greater lateral diffusion compared to the standard Martini 2 model (Figure 2A). Least squares fitting of individual ReLPS molecules from different time points within the trajectory revealed greater conformational deviation of the fast ReLPS molecules compared to the standard model. Visual inspection indicated that while there is greater conformational lability in all regions of fast Martini 2 ReLPS molecules compared to the standard models (Figure 2B), the difference appeared to be greater in the Kdo sugars. Overall, individual molecules of the fast Martini 2 ReLPS model exhibited a wider RMSD distribution compared to their standard Martini counterpart and reference atomistic trajectory analyzed as a pseudo-Martini 2 simulation (Figure S14A–C). Remarkably, RMSD distributions analyzed by picking random ReLPS molecules from the pseudo-Martini 2 simulation showed that variance within each molecule was smaller compared to either Martini 2 model. However, a larger distribution was found when comparing their means (e.g., spanning values from 0.5 to 0.9 nm). Furthermore, cation interactions with the negatively charged beads of the fast ReLPS model were weakened compared to those with the standard model (Figure S15), thereby reducing rigidity between different LPS molecules and aiding in their individual flexibility and lateral mobility. Thus, in summary, the goal of greater motion (both lateral and within the conformations of individual molecules) of ReLPS is realized in the new fast Martini 2 model. Interestingly, the Martini 3 ReLPS shows similar conformational lability to the fast Martini 2 model.

The MSDs for Martini 3 are discussed later in this study (and plots are provided in Figure S22) when we compare systems containing proteins—these systems are the same size for all three ReLPS models and thus provide a better comparison of the MSD of all three models.

**Figure 3:**
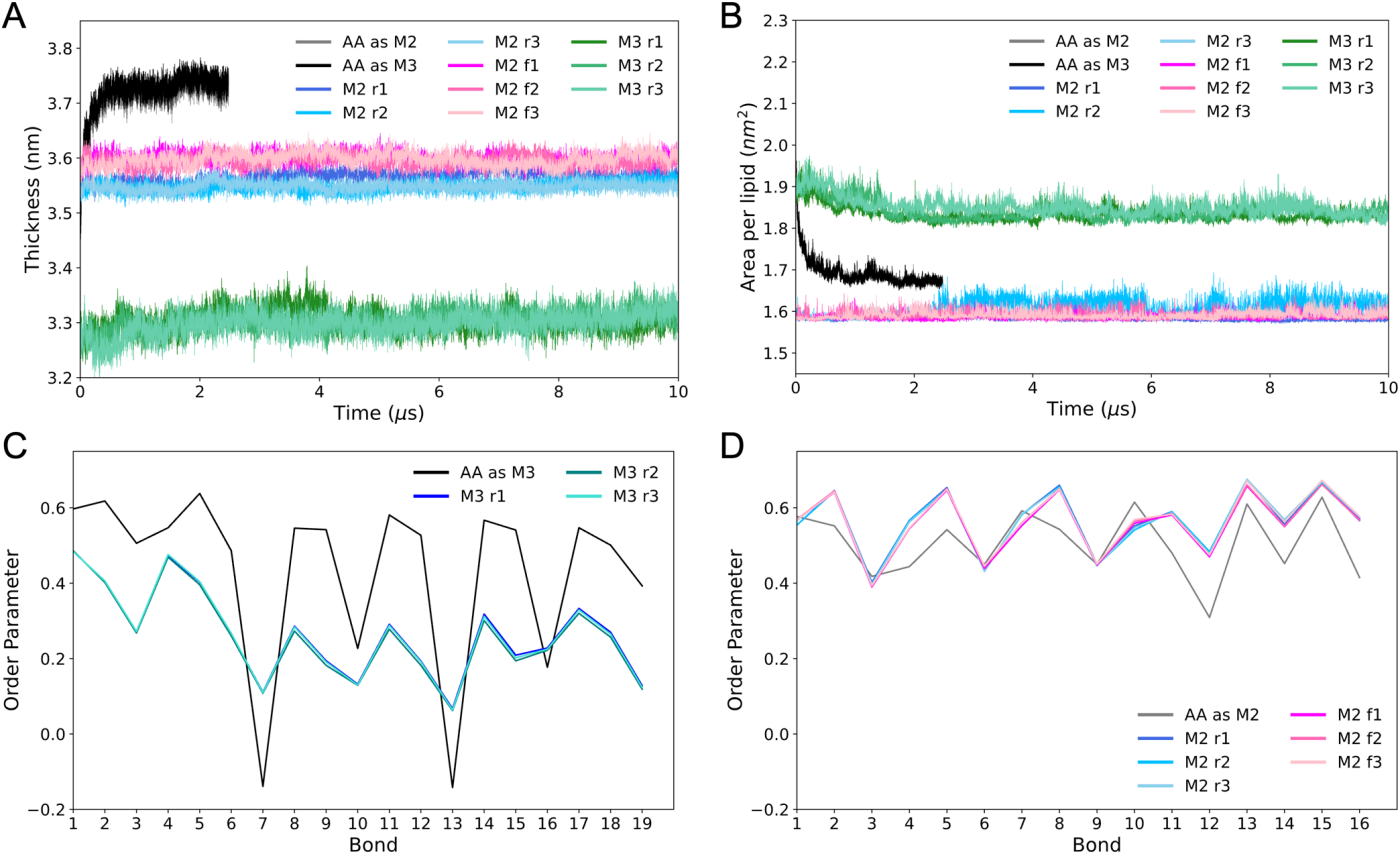
Physical properties of ReLPS in standard and fast Martini 2 and in Martini 3. (A) Membrane thickness (the curves for AA as M2 and AA as M3 are almost completely indistinguishable), (B) area per lipid (the curves for AA as M2 and AA as M3 are almost completely indistinguishable), and (C, D) lipid order parameters of our new Martini 3 ReLPS model and for the Martini 2 standard and fast ReLPS model, respectively. The plots show data taken from 3 simulation replicas with the Martini 2 standard (M2 r1–r3), Martini 2 fast (M2 f1–f3), and Martini 3 (M3 r1–r3) models and atomistic simulations mapped to Martini 2 (AA as M2) or mapped as Martini 3 (AA as M3).

As a thorough comparison, we quantified the biophysical properties of symmetric ReLPS bilayers using atomistic (CHARMM36m) and coarse-grained (Martini 2, fast Martini 2, and Martini 3) force fields. The membrane thickness, APL, and lipid order parameters (Figure 3) and partial density profiles (Figure S16) of the fast Martini 2 ReLPS showed good agreement with the standard Martini 2 and atomistic models. Both Martini 2 models give values of within 4–5% of the values obtained from the atomistic simulation but mapped into Martini 2 for both thickness and APL. The Martini 3 model showed greater deviation from atomistic models (with 9% of the APL and 11% of the thickness when the atomistic trajectories are into Martini 3). Interestingly, we note that water penetration into the all-atom ReLPS bilayer matches more closely to the Martini 3 ReLPS bilayer density than it does to either of the two Martini 2 data sets (Figure S16).

### 3.3 Testing the Permeability of ReLPS with Octane, DPC, DOPC, and PMB1

Having achieved increased mobility in the fast ReLPS model, we next sought to characterize the permeability of membranes containing these modified lipids and to compare them with the standard Martini 2 and newly parametrized Martini 3 models. The concern is that the greater mobility may render the membranes too permeable. To this end, we explored the permeation of DPC, octane, and the antibiotic polymyxin B1 (PMB1) via equilibrium MD simulations. PMB1 is used as a permeabilizing agent for LPS-containing membranes but has previously been shown (via atomistic and CGMD simulations) to be susceptible to kinetic barriers to permeation.^6−8^

**Figure 4:**
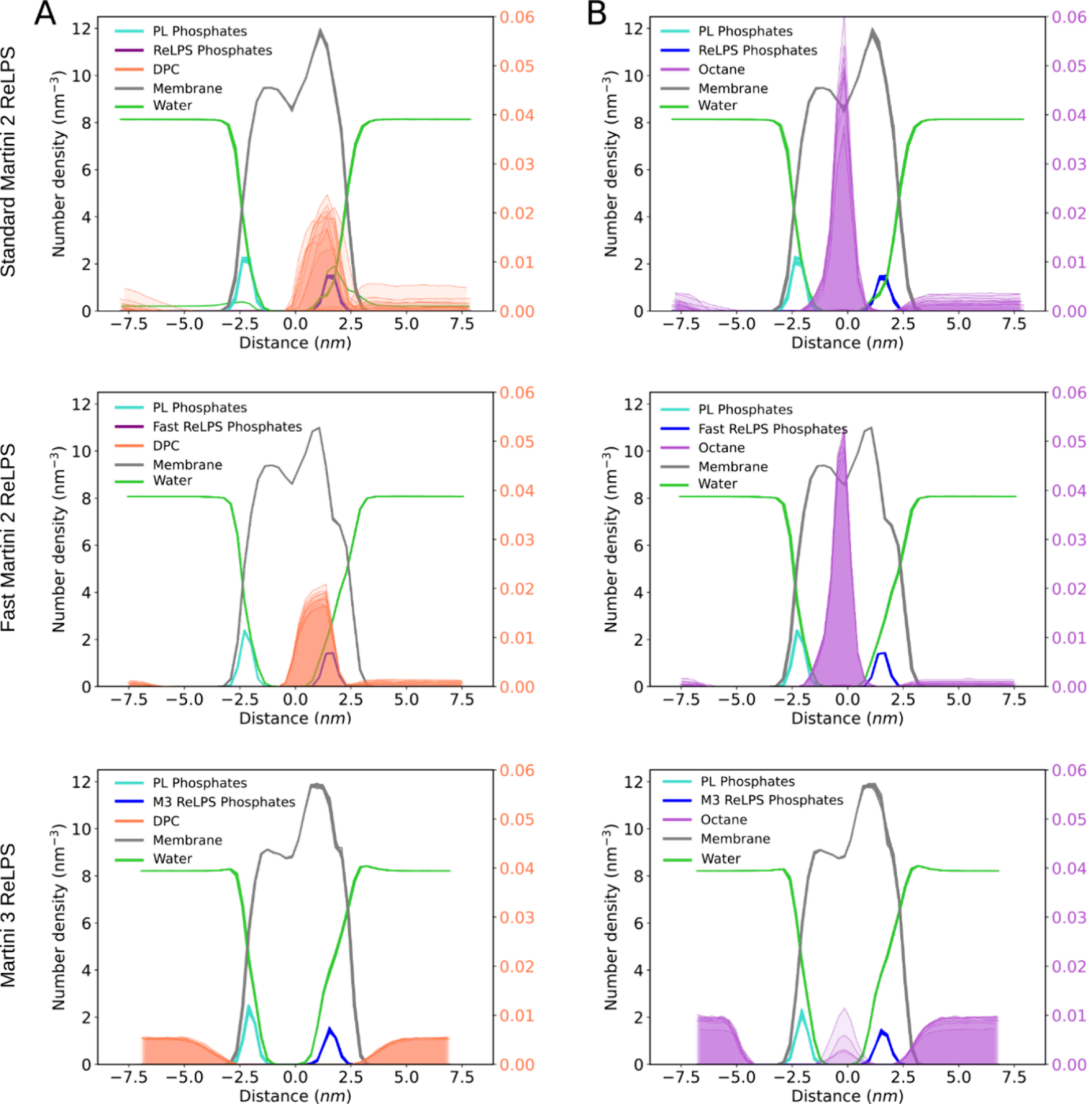
Permeability of ReLPS to DPC and octane. Number densities computed along the bilayer normal are shown in the transparent surface for DPC (A) and octane (B) in standard Martini 2 (top), fast Martini 2 (middle), and our Martini 3 model (bottom). In each of the systems containing DPC, one DPC molecule was simulated. In each of the systems containing octane, 4 octane molecules were simulated. Number densities for the phosphates of the inner leaflet (PL Phosphates) or outer leaflet (ReLPS Phosphates) and all the lipid molecules in the membrane (Membrane) are also depicted to aid the analysis. Each plot shows data computed for 10 independent 10 μs-long simulation replicas. The axis labels on the right correspond to the number densities from DPC (A) or octane (B), and a different scale was used, given the much smaller numbers compared to the other system components.

We used membrane models with 100% of either Martini 2, Martini 2 fast, or Martini 3 ReLPS in the outer leaflet and 100% PL in the inner leaflet. Octane was able to insert easily into the Martini 2 models, with all 40 octane molecules (4 molecules in 10 simulation replicas) inserting into both the standard and fast Martini 2 membrane models. In contrast, in the Martini 3-containing membrane, only 4 out of 40 octane molecules were inserted. A similar trend is observed for DPC, but in this case, we did not observe any penetration into the Martini 3-containing membrane at all. This is shown quantitatively by measuring the number density of each system component as a function of position along the *Z* dimension (perpendicular to the membrane plane) (Figure 4). We also tested the ability of a phospholipid, DOPC, to insert into the LPS leaflet (10 × 10 μs each). Insertion of DOPC was observed in 8 out of 10 simulations of the standard Martini 2 model, 7 out of 10 simulations of the fast model, and none in the Martini 3 model (Figure S17A). This indicates that our fast ReLPS model, while increasing the lateral movement of lipids, does not render the bilayer oversusceptible to permeation by small molecules, given that the permeation behavior is very similar to the Martini 2 model. Our data reveal that the Martini 2 ReLPS gives the more expected insertion behavior (compared to Martini 3); we would expect a simple detergent molecule and a phospholipid to insert into the outer membrane. We note here that there are reports in the literature about oddities in the membrane/lipid behavior of Martini 3, for example, that Martini 3 protein–water interactions need to be scaled down to successfully allow (i) phospholipid bilayer self-assembly around transmembrane protein dimers^27^ and (ii) monomeric peptides to maintain a transmembrane state.^32^ In other words, the protein–water interactions had to be made less polar to enable insertion of the protein (Glycophorin A). This is consistent with our findings here that DOPC and DPC do not penetrate into the Martini 3 ReLPS-PL bilayers, because they prefer to be in the aqueous region, i.e., the water-hydrophobic tail interaction is too attractive, just as reported for Glycophorin A by Wade and co-workers.^27^ This also explains why water penetrates further into the membrane core in the Martini 3 simulations compared to Martini 2—the water-hydrophobic tail interaction is again too attractive. We show this semiquantitatively using steered MD (Figure S18). Greater force is required to pull DPC into the core of a Martini 3 ReLPS-PL bilayer compared to the Martini 2 versions, where a barrier is experienced by DPC when entering the hydrophobic core of the membrane.

**Figure 5:**
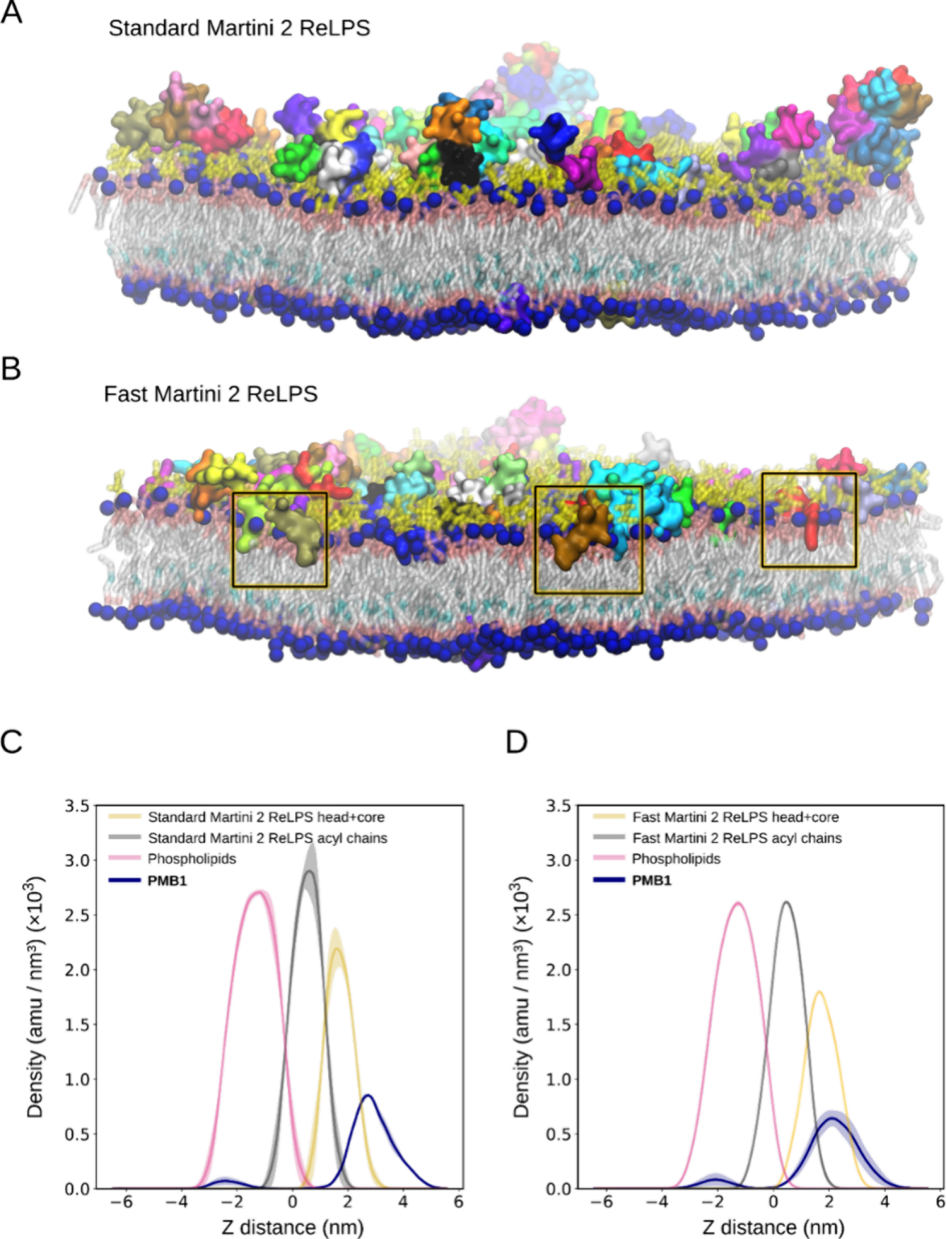
Interactions of PMB1 beads with standard and fast Martini 2 ReLPS. (A) Molecular representations of PMB1 association with the OM model containing standard Martini 2 ReLPS and (B) fast Martini 2 ReLPS in the outer leaflet at 5 μs simulation time. In (C) standard Martini 2 and (D) fast Martini 2 ReLPS-containing outer membrane models, the densities of PMB1 are shown relative to the respective ReLPS heads, tails, and phospholipids along the *Z* position of the membrane.

We next tested the penetration of polymyxin B1 (PMB1), a last resort lipoprotein antibiotic, into the ReLPS leaflet in membranes modeled with standard and fast Martini 2. While we did not observe full penetration into the ReLPS-containing leaflet of the membrane with either model, we observed deeper penetration of PMB1 with fast ReLPS compared to standard Martini 2 (Figure 5A,B and Figure S17B,C). In addition, partial density profiles along the axis perpendicular to the plane of the membrane show a greater density of PMB1 molecules embedded within the ReLPS monolayer in the fast ReLPS model. Specifically, in the fast ReLPS models, a greater number of PMB1 molecules are located at the lipid headgroup/tail interface, whereas with standard ReLPS, PMB1 is located largely at the water-headgroup interface, adhering to the membrane but not embedded (Figure 5C,D). The fact that PMB1 molecules do not fully insert into the fast ReLPS leaflet provides further evidence that this model does not cause the bilayer to be overpermeable. The absence of a Martini 3 model of PMB1 precluded the insertion study of that model.

### 3.4 Outer Membrane Protein–ReLPS Interactions

Perhaps a more stringent test of the predictive properties of our ReLPS models is their ability to identify known ReLPS binding sites on *E. coli* outer membrane proteins (OMPs). Therefore, we simulated the trimeric porin OmpF using the X-ray structure determined at 1.98 Å (PDB: 3K1B) using Martini 2 (standard and fast) and Martini 3. OmpF was embedded in a membrane containing only 2% ReLPS and 98% phospholipids in the outer leaflet to ensure accelerated lateral motion of ReLPS over 3 × 4 μs simulations.

**Figure 6:**
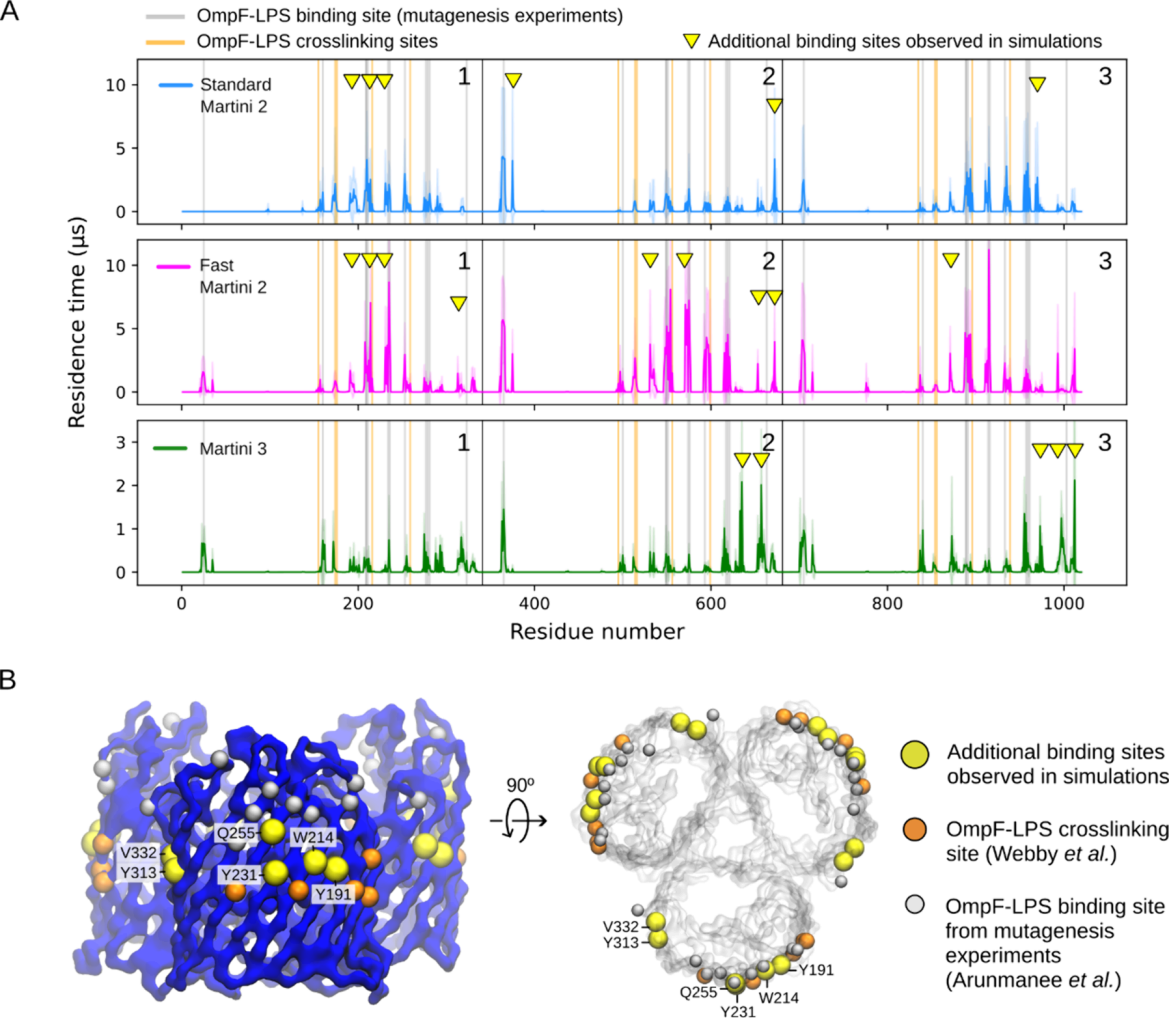
Residence times of fast and standard Martini 2 ReLPS and our Martini 3 ReLPS with OmpF compared with experimental data on LPS binding sites of OmpF. (A) Standard (blue) and fast Martini 2 ReLPS (magenta) and our Martini 3 ReLPS model (green). Gray lines indicate the residues that are proposed to contribute to LPS binding by mutagenesis experiments.^34^ Orange lines indicate OmpF-LPS cross-linking sites.^12^ The yellow triangles indicate newly identified OmpF residues from simulations whose residence times with ReLPS are comparable to or larger than those that align with experimental data. The *y*-axis for the Martini 3 model is magnified for visual clarity of its residence time peaks. (B) Side view (left) and top view of OmpF (right) with the newly identified residues shown as yellow spheres and labeled.

We calculated the residence time, i.e., the lifetime of ReLPS contacts with individual OmpF residues using PyLipID.^33^ This showed good agreement with the experimentally known LPS binding sites from two independent studies (Figure 6A), one in which LPS-binding OmpF residues were proposed based on mutagenesis experiments and on the X-ray structure of the homologous protein OmpE^34^ and the other from cross-linking studies.^12^ The mutagenesis experiments showed that mutations of positively charged residues (K25Q, K160Q, K209Q, K210Q, R235Q, K253Q, K277Q, K279Q, K281Q, and K323Q) reduced the amount of LPS bound to OmpF, whereas cross-linking experiments highlighted V155, V174, G176, T216, and L259 (referred to as V177, V196, G198, T238, and L281 by Webby et al.) to be involved in LPS binding. Our simulations across all Martini models of ReLPS were able to reproduce these data and further quantify these interactions with OmpF, thereby suggesting “hotspots” for LPS binding.

In addition to these simulations revealing ReLPS binding sites in agreement with experimentally determined LPS binding sites, we consistently observed other residence time peaks non-uniformly on all the OmpF monomeric subunits with all the CG models (Figure 6A). We note that the pattern of interactions differs between OmpF monomers due to the low concentration of ReLPS used in the simulations. On the other hand, a higher number of ReLPS molecules in the leaflet could lead to LPS–LPS aggregation and reduce sampling protein–LPS interactions. We observed that the fast Martini 2 ReLPS model showed the highest residence time peaks on average followed by the standard Martini 2 model and then the Martini 3 model (note the magnified *y*-axis for Martini 3 in Figure 6A). Our fast model exhibits higher residence time compared to the standard model as it experiences less LPS–LPS aggregation in the membrane before it associates with the protein due to the scaled-down LPS–LPS interactions. Therefore, compared to the standard model, it forms contacts with OmpF at an earlier point in time. Further, upon association of a fast ReLPS molecule with OmpF, the contacts between its 6 acyl chains and OmpF (whose nonbonded potentials have not been scaled at all) form long-lasting interactions similar to the standard model.

To aid in visualizing the residence times of the three Martini models, we fitted the coordinates of the OmpF backbone along the *x*–*y* plane and plotted the density of the center of mass of ReLPS molecules for each individual simulation (6 × 12 μs) (Figure S19B). The less localized density of the Martini 3 ReLPS molecules around the OmpF trimer clarified that they interacted with the protein but did not stay attached for as long as the Martini 2 models. Nonetheless, similar to the fast Martini 2 model, our Martini 3 model did explore and interact with a larger surface area of the protein compared to the standard Martini 2 model, which turned out to be restricted to certain areas on the protein once bound.

To assess the overall spatial localization of ReLPS with respect to the OmpF trimer, we computed their radial distribution functions (RDFs) with reference to the OmpF trimer and observed an enrichment of fast Martini 2 ReLPS compared to the standard model (i.e., the first peak, although located in the same position in the fast model, is twice than that of the standard model; Figure S19A).

We then sought to do a simple contact analysis between OmpF residues and ReLPS molecules, which matched the residence time peaks and confirmed that our simulations not only reproduced experimentally determined LPS binding sites but also predicted additional sites on OmpF that form favorable interactions with ReLPS, specifically residues Y191, W214, Y231, Q255, Y313, and V332 (Figure S20). The locations of these residues are shown on the OmpF backbone structure alongside those of LPS binding sites suggested by experiments in Figure 6B.

In addition, we calculated the number of contacts versus time of all the ReLPS models with OmpF (Figures S21 and S22) across all simulations to check if protein–ReLPS interactions are dynamic, which is essential to ensure the interactions we observe are not dependent on the initial system configuration. From these data, we calculated the number of dissociation events from OmpF of each ReLPS molecule in each simulation replica across all Martini models. To do this, we considered an ReLPS to be in contact with OmpF when there were ≥30 pairs of contacts between OmpF beads and ReLPS beads, and dissociation was then deemed to occur when there were zero contacts between the two. Taking into consideration all simulation replicas and all ReLPS molecules in each of them, we determined that our Martini 3, fast Martini 2, and standard Martini 2 models undergo a total of 1029, 43, and 67 dissociations from OmpF, respectively. This is consistent with residence time (Figure 6), RDF and 2D density (Figure S19), the number of contacts per OmpF residue (Figure S20), and the number of protein–ReLPS contacts versus time (Figures S21 and S22) analyses across all Martini models of ReLPS, which indicate that our Martini 3 model interacts with the protein more transiently compared to the Martini 2 models at this LPS concentration. Nonetheless, all Martini ReLPS models are able to dynamically bind OmpF throughout the trajectory and reproduce experimental LPS-binding sites on the surface of OmpF.

**Figure 7:**
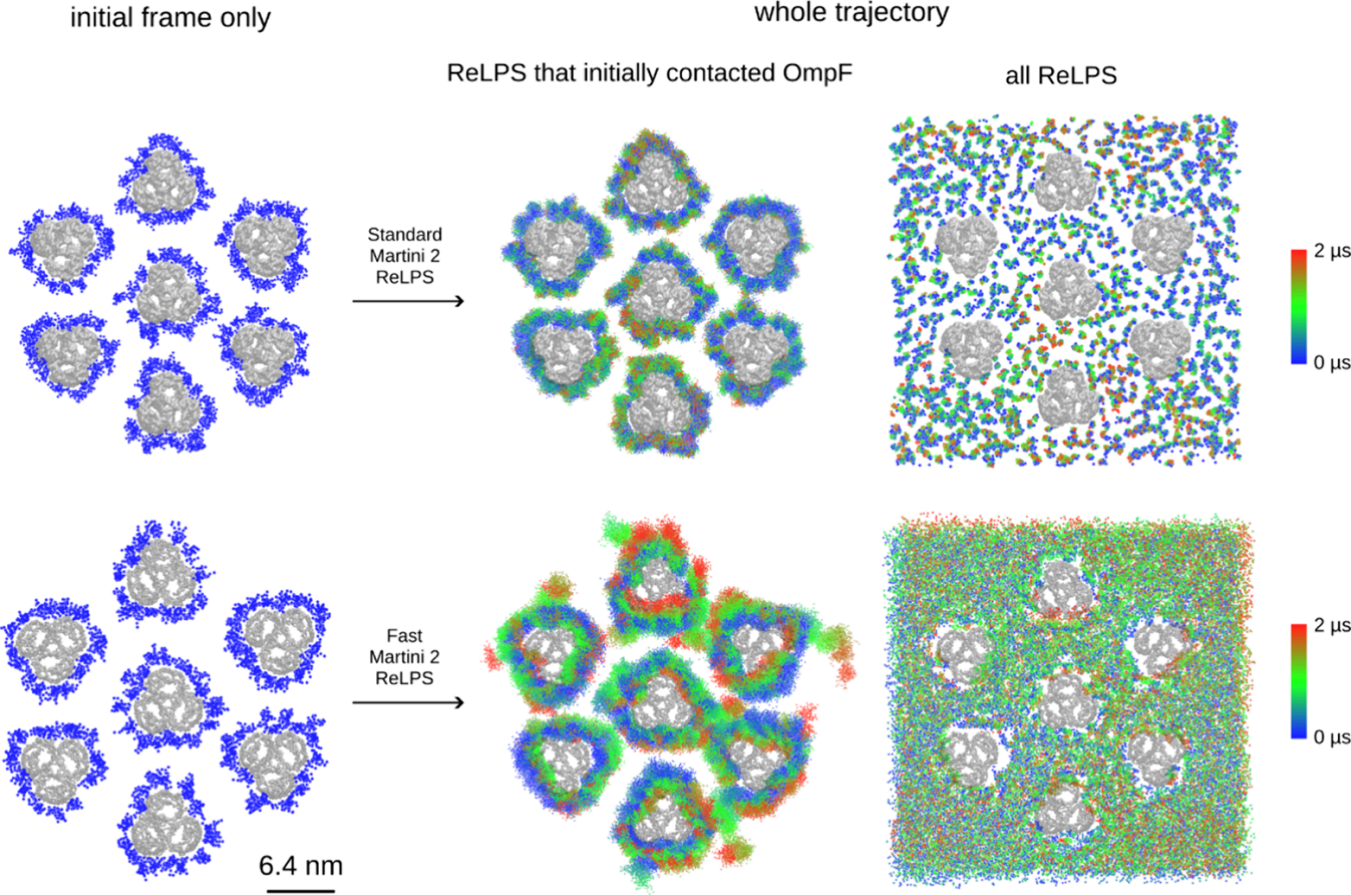
ReLPS lateral mobility in an outer membrane model system containing 7 OmpF trimers. Individual ReLPS molecules were tracked along the full trajectory (2 μs) after protein fitting. Panel (A) shows the mobility of standard Martini 2 ReLPS, whereas panel (B) is the same as panel (A) but for fast Martini 2. The middle image in each row shows the respective movement of ReLPS that was initially in the vicinity of the protein. The right panel shows the tracked path of one bead of each ReLPS in the systems, colored by time, clearly showing that fast ReLPS has increased mixing rate.

We next performed simulations of outer membrane systems containing 7x OmpF trimers (100% ReLPS in the outer leaflet) with standard and fast Martini 2 ReLPS (Figure 7A,B, respectively). In these simulations, fast ReLPS molecules that were initially in contact with OmpF explored larger membrane surface area compared to its standard Martini counterpart. Not only did fast ReLPS diffuse more, but we also observed multiple instances of ReLPS-OmpF dissociation. Moreover, global ReLPS mixing was increased in the fast ReLPS simulations compared to those containing standard ReLPS, as revealed by tracking the lateral movement of one phosphate bead (PO1) of each individual ReLPS molecule.

### 3.5 Distribution of OMPs within the Outer Membrane

**Figure 8:**
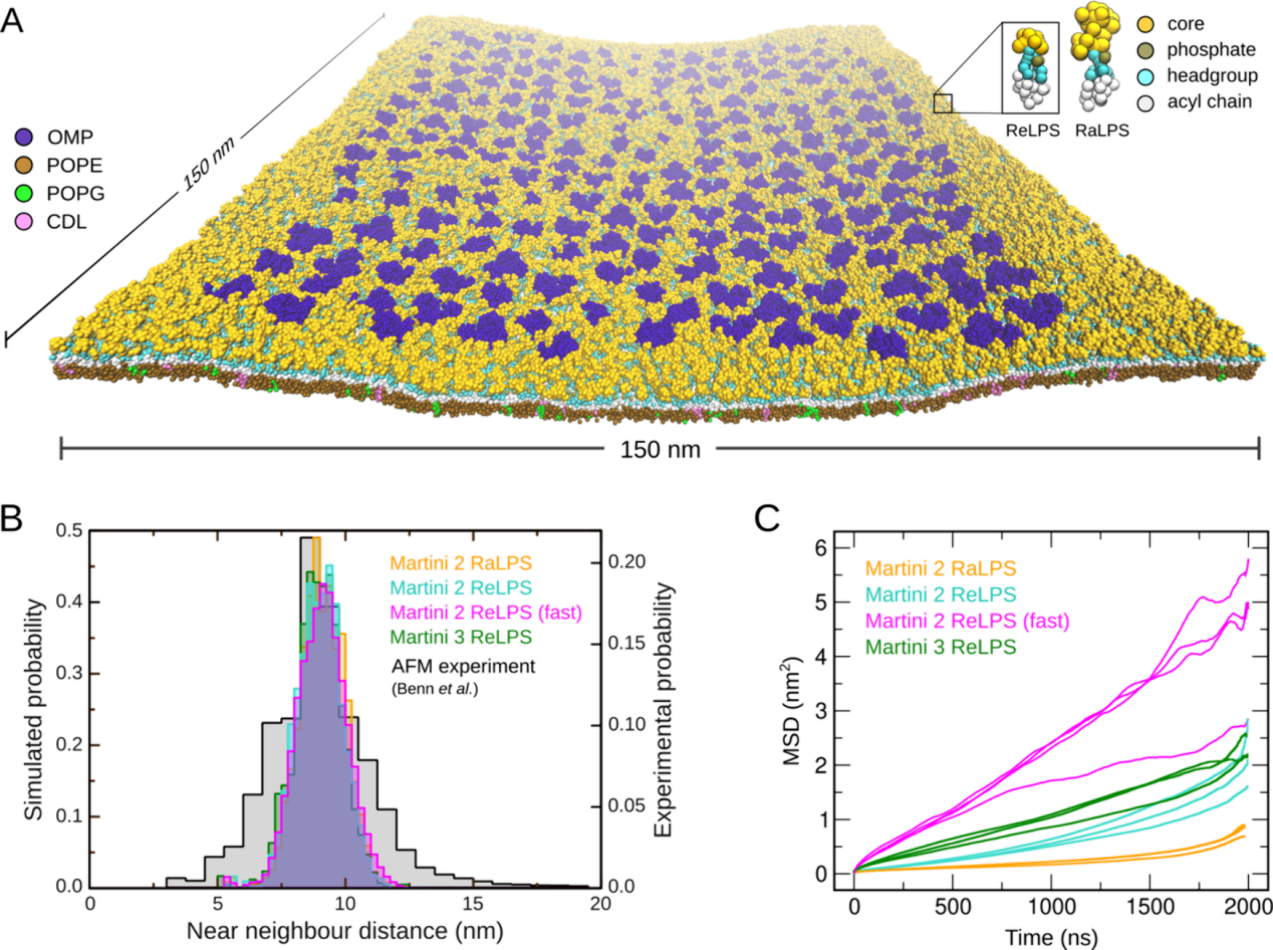
Distribution of OMPs within outer membrane models containing standard or fast ReLPS models from Martini 2 and 3 and the standard Martini 2 RaLPS model. (A) Snapshot of the OMP island embedded in a Martini 2 ReLPS-containing outer membrane model. Near-neighbor distances were calculated from this OMP island model. (B) Near-neighbor distance distribution of OMPs shown as a histogram to enable comparison between our simulated ReLPS models and AFM data. The AFM data are based on experiments conducted by Benn et al.^11^ (C) Lateral MSDs of OMPs are plotted for all three individual replicas of each simulated system. For reference, the MSD of the ReLPS molecules in the OMP island simulations from each of the three ReLPS models is provided in Figure S23. For fast ReLPS, there are four curves as one of the original three had rather low MSD (albeit within the linear region up to ca. 500 ns, agreeing well with the others), which necessitated a fourth simulation.

We have recently reported simulations of a patch of an “OMP island” as observed in AFM images using standard Martini 2 RaLPS. Our simulated model, whose near-neighbor distance was consistent with AFM data,^11^ provided the most realistic molecular simulation model of an *E. coli* OMP island to date.^12^ This model contained 7 different OMPs including multimeric proteins such as the BAM complex (in the ratio OmpF trimer:BtuB:FhuA:FhuE:FepA:LptDE:BAM = 102:20:20:19:19:19:19), giving an OMP island model spanning 150 nm^2^ along the membrane plane (Figure 8A). Here, this OMP island provides a useful test of the ability of our new ReLPS models to maintain the spatial arrangement of OMPs. Given that OMP packing into hexagonal lattices has been observed in reconstituted 2D arrays,^35,36^ in purified outer membranes,^37^ and in living bacteria,^11,38,39^ we would expect the organization of the island to be maintained in ReLPS-containing outer membrane models.

We simulated four new systems, in which the RaLPS from our original work^12^ was replaced with the standard Martini 2 ReLPS, fast Martini 2 ReLPS, and Martini 3 ReLPS models. Note that RaLPS has a longer glycan core (39 coarse-grained particles) compared to ReLPS (10 coarse-grained particles) (shown in Figure 8A). The distribution of near-neighbor distances of OMPs in the simulated OMP island (3 × 2 μs simulations of each system) was maintained, showing good agreement with the AFM data and the original previously reported Martini 2 RaLPS simulations (Figure 8B). Furthermore, the near-neighbor distance distribution between the different ReLPS models also agrees, confirming that our fast Martini 2 and new Martini 3 ReLPS models are able to reproduce the behavior of the standard Martini 2 model even in much larger systems. Meanwhile, by calculating the MSDs of OMPs, we show that the OMPs achieved varied magnitudes of lateral diffusion between the different ReLPS models. Here, we observed that the MSD of proteins embedded within the membrane containing fast ReLPS is enhanced (Figure 8C), while our fast Martini 2 ReLPS model itself has a greater MSD compared to the other Martini LPS models included in this study (Figure S23). The lowest MSD of OMPs is when they are in the Martini 2 RaLPS-containing OM model, which is unsurprising given the fact that (1) the OMPs are able to form a greater number of interactions with RaLPS molecules compared to the ReLPS models, owing to the longer length of the oligosaccharides in the former, and (2) the MSD of RaLPS itself is lower than the ReLPS model (Figure S23).

We note here that the distribution of near-neighbor distances is wider in the AFM data (Figure 8B) compared to the distributions from simulations likely due to the AFM images being taken from the surface of an entire cell, whereas the simulations only incorporate a single “OMP island”, albeit containing 218 OMPs. Overall, our data show that our new ReLPS models are able to maintain experimentally determined OMP distributions within LPS-containing outer membrane models.

## 4 Discussion

Here, we have sought to address the need for faster kinetics within LPS-containing model membranes as well as to complete the Martini 3 *E. coli* LPS modeling toolkit. Our newly parametrized Martini 3 ReLPS model is compatible with existing Martini 3 models of other lengths of LPS molecules, and we note here that there is no reason why it cannot be used in combination with other Martini 3 models for simulations of LPS molecules of varying lengths despite our model using a slightly different mapping scheme.^19^ Overall, our Martini 3 ReLPS shows agreement with its atomistic and Martini 2 counterparts and also serves to highlight the utility and compatibility of PyCGTOOL in developing bonded parameters for Martini 3. We note that there is some deviation in the area per lipid and membrane thickness of the Martini 3 ReLPS model from the Martini 2 versions, while some of these differences may arise due to general differences between the two force fields, and further work is needed to fully ascertain the origins of these differences and whether they have any functional consequences. There is greater water penetration toward the bilayer core in the Martini 3 ReLPS bilayer (and this better matches all-atom behavior), which may cause the larger APL values for the Martini 3 model, as the water interdigitates more between the lipid headgroups. It is also noted that these differences are within 5 and 9% of the atomistic APL reference value for the Martini 2 models and Martini 3 model, respectively (obtained from the coarse-grained pseudotrajectory, i.e., the atomistic trajectory mapped to their respective Martini model). These deviation ranges are similar to previously reported values for the original Martini 2 model (4–5%)^40^ and similar Lipid A or LPS molecules reported by Vaiwala and Ayappa^19^ (2–4%), albeit the calculation of the APL in the latter was computed in a different manner, i.e., dividing the surface of the membrane by the amount of lipids present in one leaflet.

Our fast Martini 2 model is stable over long simulations not only in symmetric bilayers but also in more complex model membranes, including in the presence of embedded proteins. Furthermore, this model not only reproduces the biophysical properties of the standard model very well (membrane thickness, APL, and lipid tail order parameters as shown in Figure 3), but it also shows enhanced lateral diffusion as well as greater conformational freedom within individual molecules. The fast model compared to its standard Martini 2 counterpart is slightly more permeable to polymyxin B1, an antimicrobial peptide known to be able to localize into the outer membrane of *E. coli*. We have also demonstrated that the fast model is not overly permeable to water and ions as clearly observed by the lateral partial number density data (Figure S12). One key advantage of this fast Martini 2 ReLPS model is that by increasing ReLPS mobility, (i) the relative orientation of the proteins embedded in the membrane and (ii) the patterns of protein–lipid interactions are not strongly dependent on the initial configuration.

We note here that the strategy we have adopted for enhancing the kinetics of LPS is only useful and indeed warranted for simulations of molecules that are so slowly diffusing that extremely long simulation times would be required to achieve lateral motion. In this context, it is also useful to reflect on potential limitations of our models and approach. Overall, we note here that the models for fast Martini 2 ReLPS and Martini 3 ReLPS could both benefit from further testing by the computational community. The issues of penetration of hydrophobic entities into Martini 3 membranes (reported here for ReLPS-PL bilayers and also by others for phospholipid-only-containing bilayers^27^) preclude the development of a fast Martini 3 ReLPS model, until these are resolved. Given the huge library of molecular models available in Martini 2 and, in particular, the availability of long polysaccharide chain models such as those required for longer LPS molecules including those that incorporate O-antigen, as well as ongoing issues with water–protein interactions^27,32^ and the membrane insertion oddities noted here in Martini 3, while not being the latest Martini parameter set, Martini 2 is likely to continue being the Martini force field of choice for bacterial membrane simulations for some time.^17,18^

## 5 Conclusions

We posit that the straightforward usage of our ReLPS models within the GROMACS package is a clear benefit for potential users. However, the user should carefully consider the scientific question they are addressing when using the fast ReLPS model, since its molecular chemistry is slightly altered due to the scaling of its original charges. While we are aware that algorithmically enhanced sampling methods such as temperature replica exchange molecular dynamics (T-REMD) can be used to extend the sampling of phase space even in the NPT ensemble, for LPS, they would require impractical computational resources. Additionally, as we have previously shown via Hamiltonian replica exchange, LPS mixing requires long simulation times to potentially converge.^5^ Therefore, despite its tweaked chemistry, our model offers a practical and “off the shelf” solution for simulations of ReLPS-containing bilayers.

## Supporting Information Available

Martini 3 and fast Martini 2 parameters of ReLPS (ZIP)
Additional details of the coarse-grained and atomistic simulation systems and protocols, details of simulation analyses and visualization, additional comparative analyses of the ReLPS models discussed here, and details of how the fast ReLPS was parametrized (PDF)
