# Supplementary material for: Faster but Not Sweeter: A Model of Escherichia coli Re-level Lipopolysaccharide for Martini 3 and a Martini 2 Version with Accelerated Kinetics
Source: J Chem Theory Comput. 2024 Jul 15;20(15):6890–903. doi: 10.1021/acs.jctc.4c00374 (PMC11325540; doi:10.1021/acs.jctc.4c00374)
Supplement: Supplementary file 2 — ct4c00374_si_002.pdf [file ct4c00374_si_002.pdf]

Supplementary information

Faster but not sweeter: A model of *E. coli* Re-level lipopolysaccharide for Martini 3 and a Martini 2 version with accelerated kinetics

Astrid F. Brandner [1*], Dheeraj Prakaash [1*], Alexandre Blanco González [1,2,3,4], Fergus Waterhouse [1] and Syma Khalid [1**].

1. Department of Biochemistry,
   University of Oxford
   Oxford
   OX1 3QU

2. Departamento de Física Aplicada,
   Universidade de Santiago de Compostela,
   Spain

3. Departamento de Química Orgánica,
   Universidade de Santiago de Compostela,
   Spain

4. MD.USE Innovations S.L.,
   Edificio Emprendia, 15782 Santiago de Compostela,
   Spain

* These authors contributed equally

** To whom correspondence should be addressed at: syma.khalid@bioch.ox.ac.uk

Table of Contents

1. Supplementary Methods 3
   1.1. Details for setting up the atomistic (CHARMM36m) simulation 3
   1.2. Details for setting up coarse-grained (Martini 2 and 3) simulations 3
   1.3. Simulation analyses and visualization 4
   1.4. Details of obtaining the fast ReLPS Martini 2 model 4

2. Supplementary Tables 6

3. Supplementary Figures 12

4. Supplementary References 35

1. Supplementary Methods

1.1. Details for setting up the atomistic (CHARMM36m) simulation

NPT conditions were maintained using a Nosé-Hoover thermostat [1,2], and a Parrinello-Rahman barostat [3] with a compressibility of 4.5×10^{-5} bar^{-1}. The integration timestep was set to 2 fs, and bonds to hydrogens were constrained with the LINCS algorithm [4]. Long-range electrostatics were computed using particle-mesh Ewald [5] with a tolerance of 10^{-6}, 4th-order spline interpolation, and a maximal mesh size of 0.12 nm; van der Waals interactions were shifted smoothly to zero between 1.0 and 1.2 nm.

1.2. Details for setting up coarse-grained (Martini 2 and 3) simulations

All the systems were minimized using the steepest descent algorithm until the forces converged to less than 1000 kJ/mol/nm. With the Martini 2 force field, the systems were equilibrated once at 313 K using a 20 fs timestep, except for the PMB1 simulations where a 10 fs timestep was used. The time of equilibration for simulations of the OMP island and PMB1 were 30 ns and 100 ns respectively. All other Martini 2 systems were equilibrated for 5 ns each. During equilibration, the cut-off algorithm combined with the potential-shift modifier was employed to treat both van der Waals and coulombic interactions. The van der Waals and coulombic radius was set to 1.1 nm. The pressure was maintained at 1 bar with semi-isotropic pressure coupling using the Berendsen barostat [6], the compressibility was set to 3.0-4 /bar, the temperature was maintained at 313 K using the V-rescale thermostat [7] and a time constant of 1 ps.

With the Martini 3 force field, systems were equilibrated step-wise using the Berendsen barostat for a total time of 150.6 ns i.e. (1) with 1 fs timestep at 200 K for 50 ps, (2) with 1 fs timestep at 313 K for 50 ps, (3) with 5 fs timestep at 313 K for 500 ps, (4) with 10 fs timestep at 313 K for 50 ns, (5) with 10 fs timestep at 313 K for 100 ns. From step 3 to step 5, the protein backbone and ReLPS phosphate beads were position restrained with 50 kJ/mol/nm harmonic potential. For equilibration and production stages, the reaction-field algorithm was used to treat coulombic interactions and the cutoff with the potential-shift modifier algorithms to treat van der Waals interactions in Martini 3 simulations. For the membrane permeability simulations, the octane/ DPC/ DOPC molecule was position restrained during equilibration. For steered molecular dynamics (MD) simulations, this equilibrated snapshot was used as the initial frame and a force of 1000 kJ/mol/nm was applied on the center of mass of DPC in the -Z direction to pull it from water into the bilayer using at the rate of 1 nm/ns. These steered MD simulations were performed in 5 replicas using the standard Martini 2 and Martini 3 ReLPS models. All production simulations including the steered MD simulations used the Parrinello-Rahman barostat [3] along with the velocity rescaling thermostat [7].

1.3. Simulation analyses and visualization

The GROMACS package [8] version 2019.4 or higher was used to setup, run, and post-process all simulations. PyLipID [9] was used to calculate residence times between ReLPS and outer membrane proteins (OMPs) using a lower and upper cutoff of 0.485 and 0.8 nm to define contacts. RMSD, distances, lateral diffusion, density maps, radial distribution functions and contacts were analyzed using GROMACS analysis tools: gmx rms, gmx distance, gmx msd (-lateral z), gmx densmap, gmx rdf, gmx mindist respectively. The lateral diffusion coefficients of the Martini 2 ReLPS models were obtained for independent 1 μs-long segments from a linear fit of the MSD using a time window of 20-100 ns for the fit. Area per lipid (APL) and membrane thickness were analyzed using FATSLiM [10]. Phosphate moieties were used to define the group for leaflet identification. Lipid order parameters were calculated using a modified version of the do-order-multi.py script available on the www.cgmartini.nl website (http://www.cgmartini.nl/index.php/downloads/tools/229-do-order). VMD was used for visualization of all systems [11]. NumPy [12] was used during postprocessing and analyses of data. Matplotlib [13] and Xmgrace (https://plasma- humphreygate.weizmann.ac.il/Grace/) were used for plotting. The near-neighbour (NN) index [14] was calculated using the following equation:

$$NN\ Index = (unique\ neighbour\ number - N_{NN}) / (N_{LPS} - 1 - N_{NN})$$

where unique neighbour number refers to the number of unique LPS molecules contacted by the reference LPS throughout the trajectory, N_{NN} is the number of surrounding LPS molecules in a single frame of the trajectory (taken as 6), N_{LPS} is the number of LPS molecules in the simulation.

1.4. Details of obtaining the fast ReLPS Martini 2 model

We first investigated reduction of the magnitude of the charges (by 100, 50 and 25 %) on all anionic beads of ReLPS. Each of these settings were tested with neutralizing monovalent (Na^+) and divalent (Ca^{2+}) cations. To quantify the impact of these alterations to charges we consider the following metrics: mean squared displacement (MSD), nearest neighbour index (NNI), APL, and partial densities. The NNI provides a measure of the number of neighbours encountered by an ReLPS molecule throughout a trajectory and thus gives an indication of lipid mixing. Overall, from the MSD (**fig S6A**) and NNI data (**fig S6B**), compared to the standard Martini 2 system neutralized by Ca^{2+} ions (labelled as 'Q100%, Ca^{2+}'), we observed that individual ReLPS molecules achieved greatest lateral motion when the charge of ReLPS anionic beads and cations were scaled down by 50% (labelled "Q50%, Na^+").

We then investigated why there is a maximum in the curves for MSD and NNI of ReLPS as a function of charge (**fig S6A, B**). In **fig S7A**, if we consider the RDF curves of ReLPS phosphates (PO)-Ca^{2+}, as the PO bead charge is reduced, there are fewer and fewer PO and Ca^{2+} in proximity with each other. Concomitantly, the PO-PO RDF curves reveal the reverse trend with decreasing charge. As more and more POs come closer to each other, they repel due to electrostatics leading to increased MSD and NNI. However, at the point at which the charge is totally removed from the PO bead, despite their proximity, there is no electrostatic repulsion between PO-PO beads and therefore the average MSD

and NNI drop slightly. We provide the non-bonded potential plots for each charge regime to further highlight this point (**fig S7A**). The same trend is observed with PO-Na^+ interactions (**fig S7B**).

The partial density profiles of various components of this charge-modified (Q50%, Na^+) system suggest that the system is stable and comparable with those of the standard Martini 2 system (**fig S8A**). Concomitantly, there was a minimum increase in the APL from 1.67 ± 0.23 nm^2 to 1.73 ± 0.3 nm^2 (**fig S8B**). Therefore, based on these data, the model whereby the charges of both the ReLPS anionic beads and neutralizing cations were scaled down by 50% was deemed to provide the best compromise between deviation from the standard Martini 2 model and faster kinetics, and was taken forward to the next step.

We next investigated scaling down the inter-ReLPS LJ interactions (which leads to a shallower well-depth of potential energy). We tested a variety of scaling combinations i.e. scaling the LJ interactions of only GlcN headgroup beads, only the Kdo core (S beads), and both GlcN and Kdo core beads simultaneously (**fig S9A-C**). The scaling regimes were evaluated by calculating MSD and NNI of ReLPS. These data revealed that scaling only the Kdo core had little impact on MSD and NNI, whereas greater impact was achieved when modifying either only the GlcN headgroup beads or GlcN and Kdo core beads. Thus, in the interests of limiting chemical modifications as much as possible, only alterations to the GlcN beads were considered hereon. Compared to the standard Martini 2 model, while scaling down the LJ interactions of the GlcN beads by 5 levels (as defined by Martini 2 conventions) led to the greatest lateral diffusion (**fig S9B**), we took into account the (1) change in the APL i.e. an increase less than 5% (**table S3**), (2) the partial density profiles showing a good agreement with the standard Martini 2 model (**fig S10A**), and (3) membrane thickness showing an increase (~1%) less than 0.1 nm (**fig S10B**). As a result, we considered the model with LJ scaled down by 2 levels.

2. Supplementary Tables

Table S1. ReLPS CHARMM36m (all-atom) to Martini 3 (CG) mapping scheme.

CG name	CG type	CHARMM36m names															
ZM1	SN6r	OA5	HA5	CA5	CA6	HA6S	HA6R	OA6									
ZM2	TC1	HA1	CA1	HA2	CA2												
ZM3	SN6	CA3	CA4	HA3	HA4	OA4	HOA4										
VS1	TC4	ZM1	ZM2	ZM3													
PO1	D	OA1	PA	OPA2	OPA3	OPA4											
ZM4	N4a	OA3	C31	O31	C32	H32R	H32S										
ZM5	P3	NA2	HNA2	C11	O11	C12	H12R	H12S									
CF	P1	C13	H13R	O13	HO13	C14	H14R	H14S	C15	H15R	H15S	C16	H16R	H16S			
C1F	C1	C17	H17R	H17S	C18	H18R	H18S	C19	H19R	H19S	C110	H110R	H110S				
C2F	C1	C111	H111R	H111S	C112	H112R	H112S	C113	H113R	H113S	C114	H114R	H114S	H114T			
CE	P1	C33	H33R	O33	HO33	C34	H34R	H34S	C35	H35R	H35S	C36	H36R	H36S			
C1E	C1	C37	H37R	H37S	C38	H38R	H38S	C39	H39R	H39S	C310	H310R	H310S				
C2E	C1	C311	H311R	H311S	C312	H312R	H312S	C313	H313R	H313S	C314	H314R	H314S	H314T			
GM1	TC1	CB1	CB2	HB1	HB2												
GM2	SN5r	OB5	CB5	CB6	OB6	HB6S	HB6R	HB5									
GM3	TC1	CB3	CB4	HB4	HB3												
VS2	TC4	GM1	GM2	GM3													
PO2	D	OB4	PB	OPB2	OPB3	OPB4											
GM4	SN4a	OB3	C71	O71	C72	H72R	H72S										
GM5	P3	NB2	HNB2	C51	O51	C52	H52R	H52S									
CAB	N4a	C73	H73R	O73	C81	O81											
C1A	C1	C82	H82R	H82S	C83	H83R	H83S	C84	H84R	H84S	C85	H85R	H85S				
C2A	C1	C86	H86R	H86S	C87	H87R	H87S	C88	H88R	H88S	C89	H89R	H89S				
C3A	C1	C810	H810R	H810S	C811	H811R	H811S	C812	H812R	H812S	C813	H813R	H813S	C814	H814R	H814S	H814T
C1B	SC1	C74	H74R	H74S	C75	H75R	H75S	C76	H76R	H76S							
C2B	C1	C77	H77R	H77S	C78	H78R	H78S	C79	H79R	H79S	C710	H710R	H710S				
C3B	C1	C711	H711R	H711S	C712	H712R	H712S	C713	H713R	H713S	C714	H714R	H714S	H714T			
CCD	N4a	C53	H53R	O53	C61	O61											
C1C	SC1	C62	H62R	H62S	C63	H63R	H63S	C64	H64R	H64S							
C2C	C1	C65	H65R	H65S	C66	H66R	H66S	C67	H67R	H67S	C68	H68R	H68S				
C3C	C1	C69	H69R	H69S	C610	H610R	H610S	C611	H611R	H611S	C612	H612R	H612S	H612T			
C1D	SC1	C54	H54R	H54S	C55	H55R	H55S	C56	H56R	H56S							
C2D	C1	C57	H57R	H57S	C58	H58R	H58S	C59	H59R	H59S	C510	H510R	H510S				
C3D	C1	C511	H511R	H511S	C512	H512R	H512S	C513	H513R	H513S	C514	H514R	H514S	H514T			
KR1	TC1	CK2	CK3	HK31	HK32												
KR2	TN2a	OK6	CK6	HK6													
KR3	SP1r	CK4	HK4	OK4	CK5	HK5	HKO5	OK5									
VS3	TC4	KR1	KR2	KR3													
KS1	SQ5n	CK1	OK11	OK12													
KS2	SP3	CK7	HK7	OK7	HKO7	CK8	HK81	HK82	OK8	HKO8							
KR4	TC2	CL2	CL3	HL31	HL32												
KR5	TN2a	OL6	CL6	HL6													
KR6	SP4r	CL4	CL5	HL4	OL4	HLO4	HLO5	HL5	OL5								
VS4	TC4	KR4	KR5	KR6													
KS3	SQ5n	CL1	OL11	OL12													
KS4	SP4r	CL7	HL7	CL8	HL81	HL82	OL8	HLO8	HLO7	OL7							

Table S2. Martini 3 ReLPS bonded parameters

Table S2A Bond potential parameters

I	j	b_{eq} (nm)	K_{bond} (kJ mol^{-1} nm^{-2})
18	20	0.33193	40087.22466
18	16	0.21976	14540.77476
16	1	0.34141	11331.96785
2	5	0.3876	20899.64498
2	7	0.33386	9374.37478
3	6	0.37445	5658.56784
6	12	0.26948	8527.95542
12	13	0.35298	2987.37659
13	14	0.42314	3968.52702
14	15	0.43254	3317.70632
7	8	0.28111	9956.27756
8	9	0.34939	3021.2073
9	10	0.42254	3962.00381
10	11	0.43329	3311.89181
18	21	0.36614	15885.94134
21	23	0.29614	5703.90855
23	24	0.34514	3992.80511
24	25	0.36632	4720.95029
25	26	0.36676	4874.236
26	27	0.43459	3267.08298
23	28	0.37121	2861.41583
28	29	0.41809	3880.97892
29	30	0.4869	2522.95352
16	22	0.33178	21486.34755
22	31	0.32286	5666.22972
31	32	0.34514	4202.04338
32	33	0.4207	3591.24363
33	34	0.48583	2478.43986
31	35	0.36677	2989.03568
35	36	0.41871	3819.13206
36	37	0.48636	2522.27919
17	38	0.40263	31925.8785
39	43	0.26255	16970.36192
38	42	0.29607	23851.31594
40	44	0.41495	8477.0618
44	48	0.29313	22756.97413
45	49	0.26201	31206.05154

Table S2B Constraint bond parameters

I	j	length (nm)
1	2	0.36594
2	3	0.2937
1	3	0.26594
16	17	0.37406
17	18	0.27251
16	18	0.21976
38	39	0.33719
39	40	0.23918
38	40	0.25761
44	45	0.33821
45	46	0.25799
46	44	0.24912

Table S2C Flexible bond potential parameters*

Used for flexible minimization instead of constraints

I	j	b_{eq} (nm)	K_{bond} (kJ mol^{-1} nm^{-2})
1	2	0.36594	5402.99617
2	3	0.2937	42683.97871
1	3	0.26594	15894.44095
16	17	0.37406	16155.77694
17	18	0.27251	57729.28349
16	18	0.21976	14540.77476
38	39	0.33719	46272.12583
39	40	0.23918	21246.2869
38	40	0.25761	16615.30225
44	45	0.33821	45549.01063
45	46	0.25799	33568.59692
46	44	0.24912	13902.94084

Table S2D Angle potential paramaeters

I	j	k	ang_0 (deg)	K_{ang}(kJ mol^{-1} deg^{-1})
23	24	25	134.45984	25.36745
23	28	29	127.94594	24.29019
23	25	26	142.55945	22.24468
25	26	27	143.55522	25.89231
28	29	30	147.4	24.89224
18	21	23	145.99218	500
31	32	33	133.05173	21.13936
31	35	36	129.14664	22.67202
35	36	37	147.37221	24.64123
32	33	34	146.5092	23.49165
16	22	31	132.10041	500
3	6	12	118.8385	500
12	13	14	145.84456	28.76617
13	14	15	148.17859	24.91223
7	8	9	122.7317	13.70852
8	9	10	147.17968	28.13906
9	10	11	148.28177	25.08017
5	2	1	95.52492	91.99828
20	18	17	84.60119	442.79611
17	16	22	162.00306	59.24425
3	2	7	117.64862	500
17	16	1	89.85974	218.65071
18	16	1	133.11893	500
16	1	3	103.54873	51.79553
16	1	2	99.24979	45.52129
40	38	17	95.21793	676.88135
39	38	17	52.93281	1061.04971
38	17	18	161.87875	305.40678
39	38	17	52.93281	1061.04971
38	17	16	130.94667	254.45003
46	44	40	110.89332	182.88706
44	40	38	88.2129	386.88439
45	44	40	64.83742	402.35729
44	40	39	145.59779	194.73409
49	45	46	124.27451	286.57197
48	44	45	89.56246	1244.28317
43	39	40	120.56776	148.08985
42	38	39	88.90197	1267.36651

Table S2E Dihedral potential paramaeters

I	j	k	l	dih_0 (deg)	K_{dih}(kJ mol^{-1} deg^{-1})	multiplicity
17	16	1	2	-36.67333	12.59321	1
45	44	40	38	154.24543	7.88236	1
39	38	17	18	89.92181	6.92723	1

Table S3. Choosing an LJ-modified model of Martini 2 ReLPS such that the difference between the APL of the standard Martini 2 (Head LJ+0) and of the modified Martini 2 model (Head LJ+*n*) is < 5% (n>0).

**Model**	**Head LJ+0**	**Head LJ+1**	**Head LJ+2**	**Head LJ+3**	**Head LJ+4**	**Head LJ+5**	**Fast**
**A_{LPS} (nm^2)**	1.625 ± 0.0115	1.648 ± 0.0117	1.678 ± 0.0105	1.721 ± 0.01	1.766 ± 0.00961	1.805 ± 0.00942	1.774 ± 0.2787
**ΔA_{LPS} (nm^2)**	0	0.023	0.053	0.096	0.141	0.18	0.149
**ΔA_{LPS} (%)**	0	1.42	**3.26**	5.91	8.68	11.1	9.17

Table S4. Details on box dimensions per system and GROMACS versions to perform production simulations. Box dimension units are in (X x Y x Z) nm. Note that the box dimensions are shown for 3 replicas at the end of their production simulations, including those systems which were simulated for more than 3 replicas. In the third column, all box dimensions that were setup before energy minimization are mentioned, except the cells where "eq0" is mentioned in which case box dimensions were obtained after a short equilibration. Abbreviations used in the table are: C36m: CHARMM36m, M2: Martini 2 standard, M2 fast: fast Martini 2, M3: Martini 3, OM: outer membrane.

(page 10-11)

System	Force field/ LPS type	Box dimension (before minimization)	Box dimension at end of production replica 1	Box dimension at end of production replica 2	Box dimension at end of production replica 3	Gromacs version used for production simulations
Symmetric ReLPS bilayer	C36m	15.03662 x 15.03662 x 19.41726	14.81480 x 14.81480 x 17.89610	-	-	2021.3
Symmetric ReLPS bilayer	M3	15.01471x 15.01471 x 25.00000	14.69005 x 14.69005 x 15.95020	14.65969 x 14.65969 x 16.00532	14.67592 x 14.67592 x 15.97799	2021.4
Symmetric ReLPS bilayer	M2	**eq0** 13.75540 13.75540 19.54450	13.68184 x 13.68184 x 16.49011	13.68082 x 13.68082 x 16.46491	13.69249 x 13.69249 x 16.48008	2021.4
Symmetric ReLPS bilayer	M2 fast	**eq0** 13.75540 13.75540 19.54450	13.68943 x 13.68943 x 16.46326	13.68797 x 13.68797 x 16.47290	13.69984 x 13.69984 x 16.42741	2021.4
OM + DOPC	M2	12 x 12 x 16	11.72477 x 11.72477 x 15.85321	11.72824 x 11.72824 x 15.88695	11.75022 x 11.75022 x 15.80153	2022.4
OM + DOPC	M2 fast	12 x 12 x 16	11.94381 x 11.94381 x 15.31512	11.88679 x 11.88679 x 15.40701	11.99261 x 11.99261 x 15.18811	2022.4
OM + DOPC	M3	12 x 12 x 16	12.12834 x 12.12834 x 13.77261	12.11392 x 12.11392 x 13.83019	12.19810 x 12.19810 x 13.62773	2022.4
OM + DPC	M2	12 x 12 x 16	11.69912 x 11.69912 x 15.90881	11.71885 x 11.71885 x 15.90774	11.69615 x 11.69615 x 15.93282	2022.4
OM + DPC	M2 fast	12 x 12 x 16	11.92728 x 11.92728 x 15.31141	11.96242 x 11.96242 x 15.24096	11.96738 x 11.96738 x 15.23526	2022.4
OM + DPC	M3	12 x 12 x 16	12.12295 x 12.12295 x 14.00519	12.19112 x 12.19112 x 13.85510	12.11945 x 12.11945 x 14.04823	2022.4
OM + DPC	Steered MD (M2)	12.15461 x 12.15461 x 14.8195	11.98595 x 11.98595 x 15.14625	11.99772 x 11.99772 x 15.13335	11.97858 x 11.97858 x 15.19924	2019.4
OM + DPC	Steered MD (M3)	12.16435 x 12.16435 x 13.92706	12.10633 x 12.10633 x 14.00992	12.21149 x 12.21149 x 13.79521	12.17416 x 12.17416 x 13.89193	2019.4
OM + octane	M2	12 x 12 x 16	11.58816 x 11.58816 x 16.17199	11.72339 x 11.72339 x 15.83037	11.63015 x 11.63015 x 16.14074	2022.4

OM + octane	M2 fast	12 x 12 x 16	11.97134 x 11.97134 x 15.27016	11.87766 x 11.87766 x 15.44597	11.96371 x 11.96371 x 15.25185	2021.4
OM + octane	M3	12 x 12 x 16	12.26741 x 12.26741 x 13.43850	12.11925 x 12.11925 x 13.84160	12.21953 x 12.21953 x 13.56165	2021.4
OmpF + 7 LPS	M2	16 x 16 x 14	16.53256 x 16.53256 x 12.72750	16.66357 x 16.66357 x 12.53920	16.68552 x 16.68552 x 12.49366	2022.4
OmpF + 7 LPS	M2 fast	16 x 16 x 14	16.69026 x 16.69026 x 12.49359	16.71332 x 16.71332 x 12.46103	16.70623 x 16.70623 x 12.43382	2022.4
OmpF + 7 LPS	M3	16 x 16 x 14	16.75574 x 16.75574 x 12.00178	16.74435 x 16.74435 x 12.02074	16.77810 x 16.77810 x 11.95169	2022.4
OM + 7x OmpF	M2	40 x 40 x 12	40.06454 x 40.06454 x 10.59544	40.07512 x 40.07512 x 10.63190	40.09600 x 40.09600 x 10.53331	2020.3
OM + 7x OmpF	M2 fast	40 x 40 x 12	40.78138 x 40.78138 x 10.24050	40.87509 x 40.87509 x 10.24242	40.83708 x 40.83708 x 10.14357	2020.3
OM + 7x OmpF	M2 standard + fast (50/50%) uniform	40 x 40 x 12	38.84201 x 38.84201 x 10.87231	38.84872 x 38.84872 x 10.84959	38.86150 x 38.86150 x 10.85613	2020.3
OM + 7x OmpF	M2 standard + fast (50/50%) non-uniform	40 x 40 x 12	40.10992 x 40.10992 x 10.57044	40.00848 x 40.00848 x 10.68509	39.96832 x 39.96832 x 10.60062	2020.3
OMP island	M2	150 x 150 x 30	146.07164 x 146.07164 x 27.57932	146.80037 x 146.80037 x 27.30904	146.83083 x 146.83083 x 27.29945	2022.4
OMP island*	M2 fast	150 x 150 x 30	150.12053 x 150.12053 x 26.10580	149.97099 x 149.97099 x 26.15880	150.06816 x 150.06816 x 26.13442*	2022.4
OMP island	M3	150 x 150 x 30	141.61624 x 141.61624 x 30.00586	140.52150 x 140.52150 x 30.47952	142.38239 x 142.38239 x 29.68060	2022.4
OM + PMB1	M2	20 x 20 x 12	20.2 x 20.2 x 11.8	20.2 x 20.2 x 11.8	20.2 x 20.2 x 11.8	2021.5
OM + PMB1	M2 fast	20 x 20 x 12	20.8 x 20.8 x 11.1	20.6 x 20.6 x 11.4	20.5 x 20.5 x 11.4	2021.5

*OMP island box dimensions with M2 fast ReLPS in replica 4 (extra replica): 149.89865 x 149.89865 x 26.19435 nm^3

3. Supplementary Figures

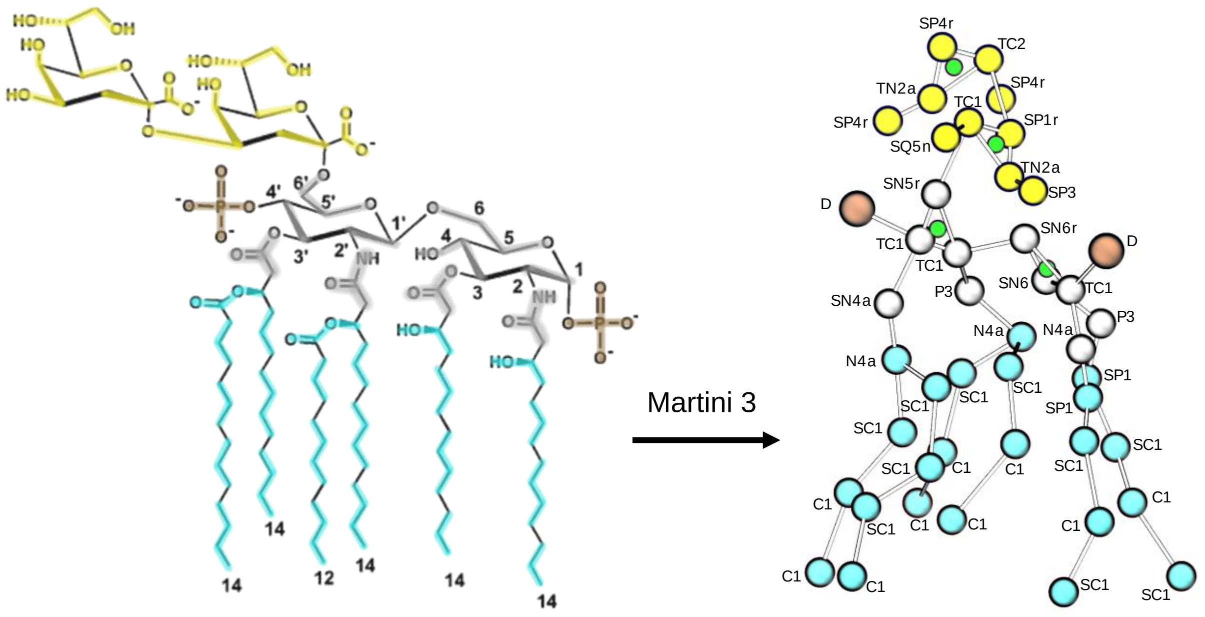

S1. Martini 3 ReLPS mapping and associated bead types. Groups of two to four heavy atoms were mapped into coarse-grained (CG) beads. Notably the sugar rings were modelled with an extra virtual site bead (depicted in green) to better characterize their volume and interactions. Here, each highlighted segment of the 2D depiction corresponds to the same-coloured bead on the right CG molecule taken from a simulation snapshot. The four different colours represent: Kdo sugars i.e. first two in core region (yellow), GlcN sugars from lipid A moiety (grey), phosphate substituents (brown), acyl chains (cyan). The labels on the CG representation show the final assigned beadtype.

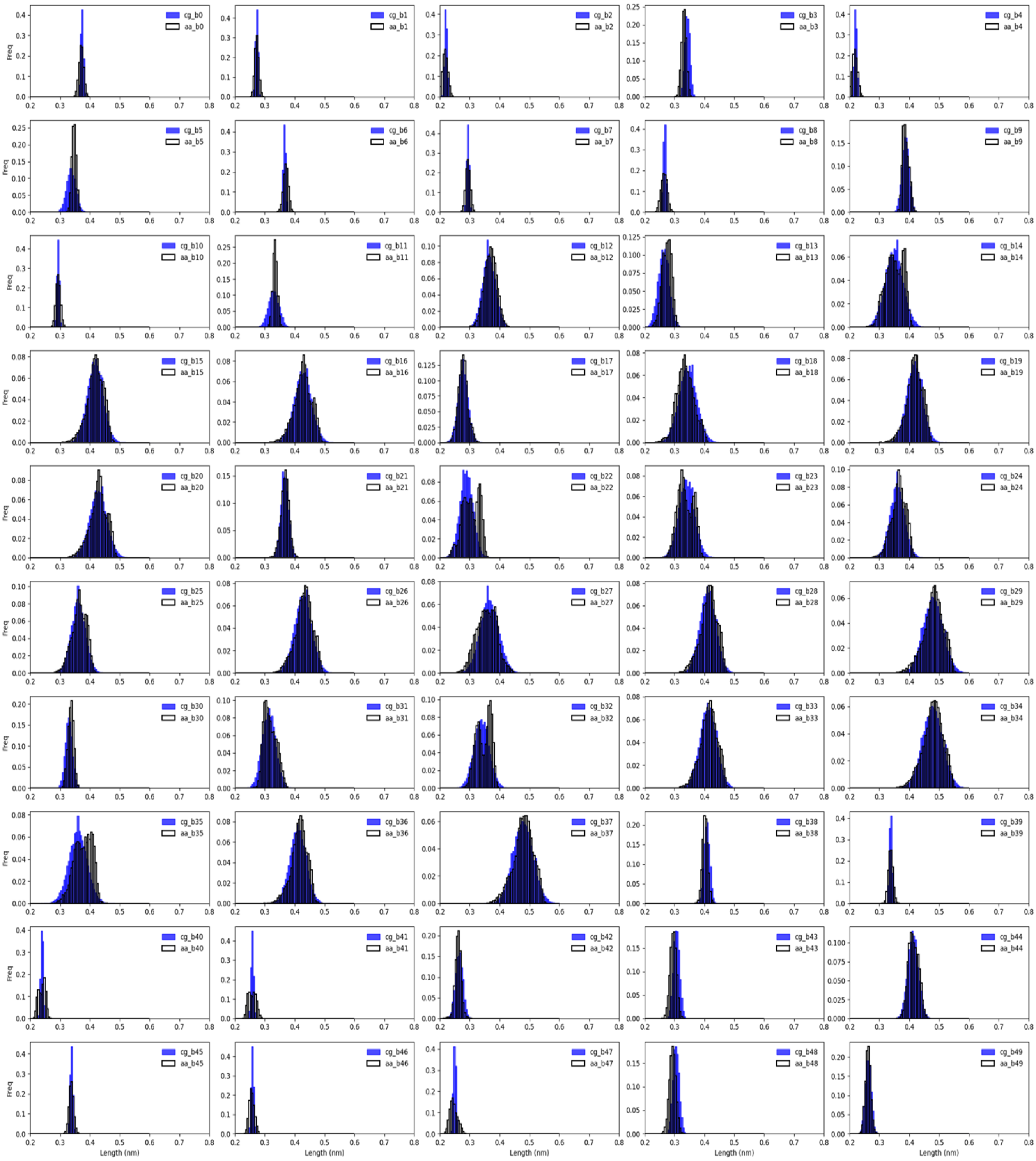

S2. Martini 3 ReLPS bond distributions. Distributions for all the bonds are plotted separately. Blue bins correspond to the Martini 3 model whereas black outlines correspond to the data obtained from pseudo-coarse-grained atomistic simulation (i.e converting the atomistic coordinates into coarse-grain model for each of the frames).

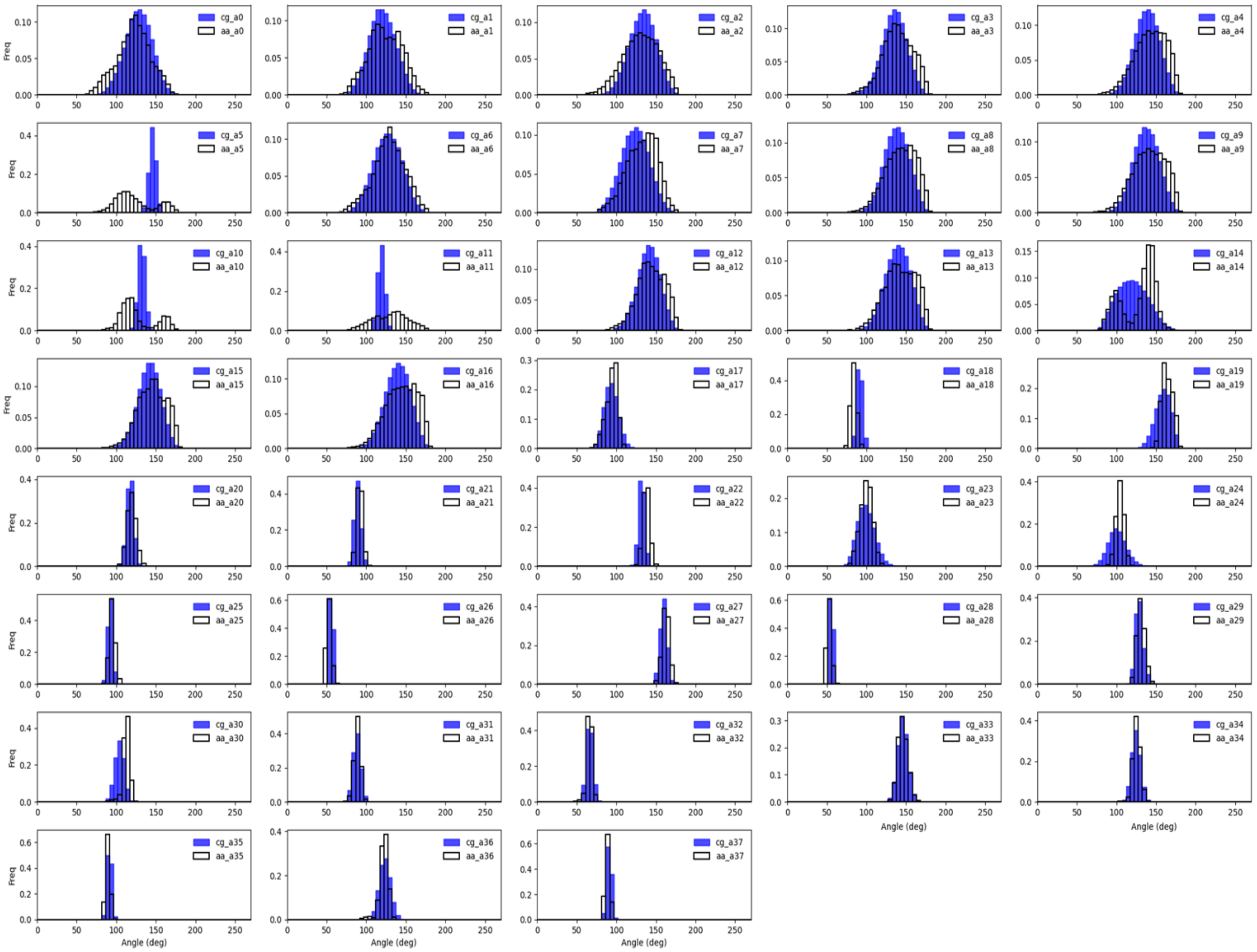

S3. Martini 3 ReLPS angle distributions. Distributions for all the angles are plotted separately. Blue bins correspond to the Martini 3 model whereas black outlines correspond to the data obtained from pseudo-coarse-grained atomistic simulation (i.e converting the atomistic coordinates into coarse-grain model for each of the frames).

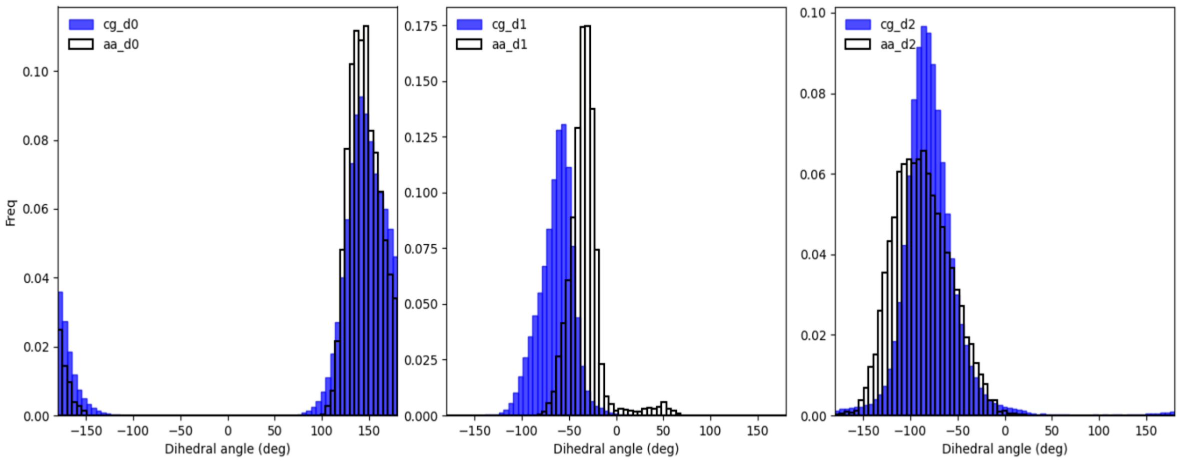

S4. Martini 3 ReLPS dihedral distributions. Distributions for all the angles are plotted separately. Blue bins correspond to the Martini 3 model whereas black outlines correspond to the data obtained from pseudo-coarse-grained atomistic simulation (i.e. atomistic coordinates converted into coarse-grained models for each of the frames).

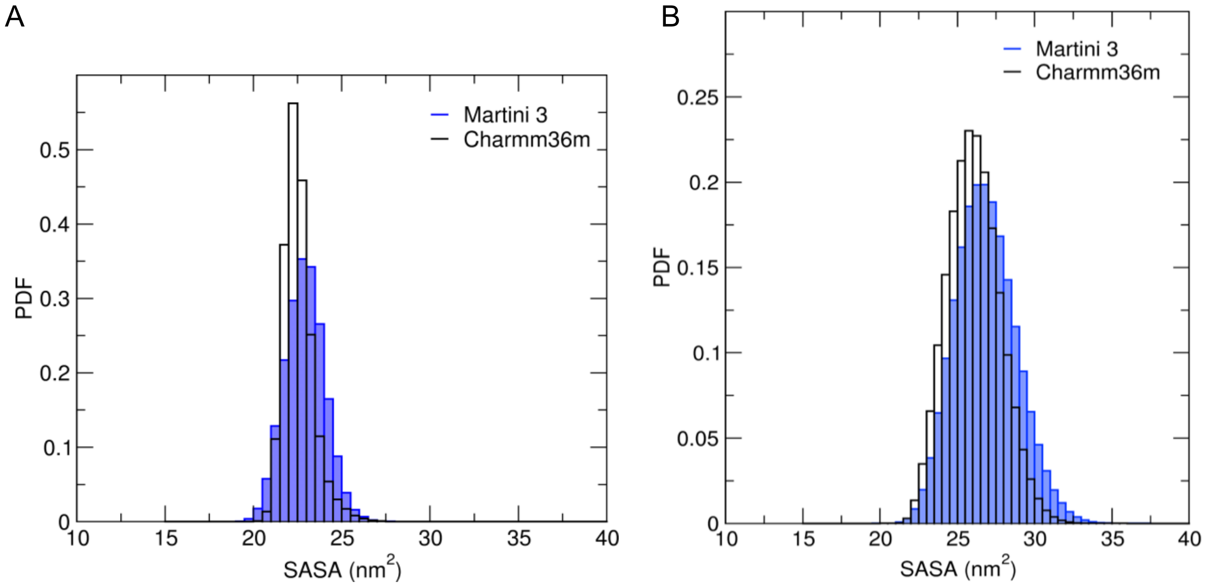

S5. ReLPS Solvent-accessible surface area (SASA) distribution. Probability distribution functions (PDF) of the SASA of a single ReLPS **(A)** in solution and **(B)** in a symmetric bilayer are plotted in blue and compared with their atomistic counterpart in black (CHARMM36m).

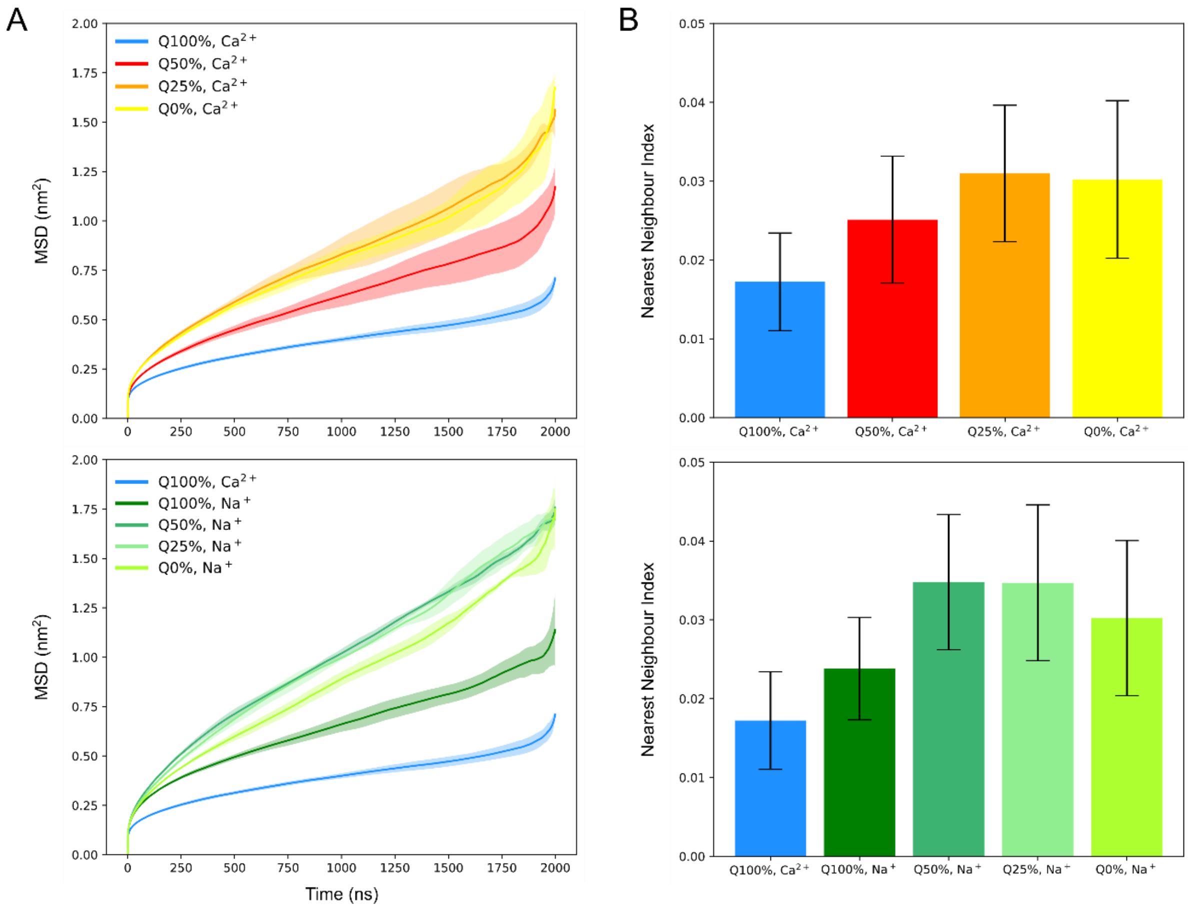

S6. Evaluating the influence of scaling down charges on Martini 2 ReLPS beads and cations on its (A) MSD and (B) NNI. For each plot, the data shown are calculated from three simulation replicas. In (A), the dark line represents the mean value from the three trajectories and the shaded regions indicate the standard deviation. Systems are labelled according to the modifications made to the anionic charges of ReLPS beads and the ions used to neutralize the system. For example, “Q50%, Na^+” means that the negative charges on all anionic ReLPS beads were scaled down to 50% and the system was neutralized by monovalent cations in addition to 0.15 M NaCl. The MSD and NNI data are split into two plots each for visual clarity.

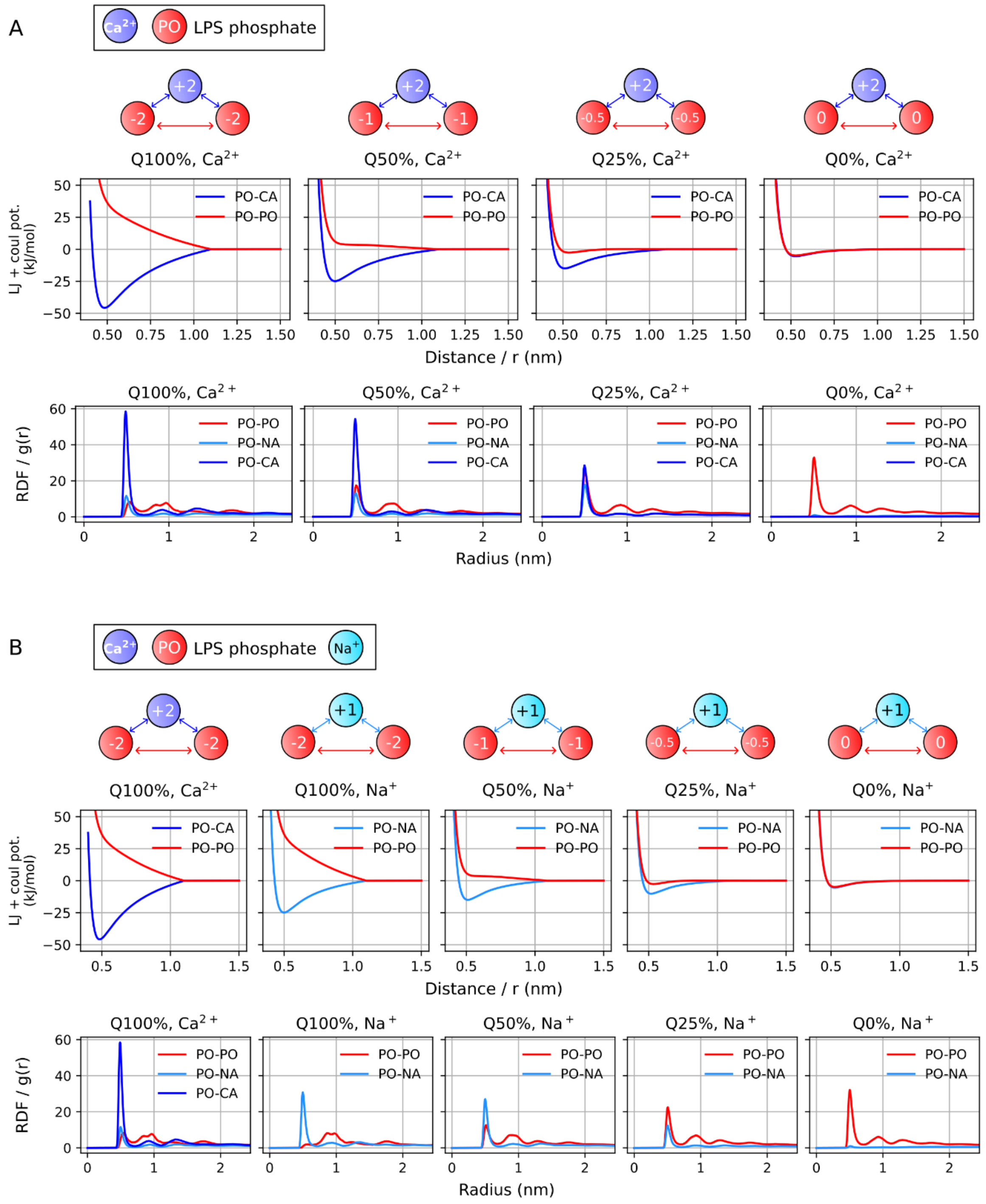

S7. Reasoning for MSD and NNI of ReLPS approaching a peak and then decreasing when their phosphate bead charges are scaled from 100% to 50%, 25% and 0%. Total potential energies (LJ + coulombic potentials) (top row) and RDFs (bottom row) of monovalent, divalent cations, and other LPS phosphates (PO beads) with reference to LPS phosphates, labelled as 'PO-NA', 'PO-CA', 'PO-PO' respectively. The system charge neutralizing cations are **(A)** the divalent cations (Ca^{2+}), and **(B)** the monovalent cations (Na^{+}).

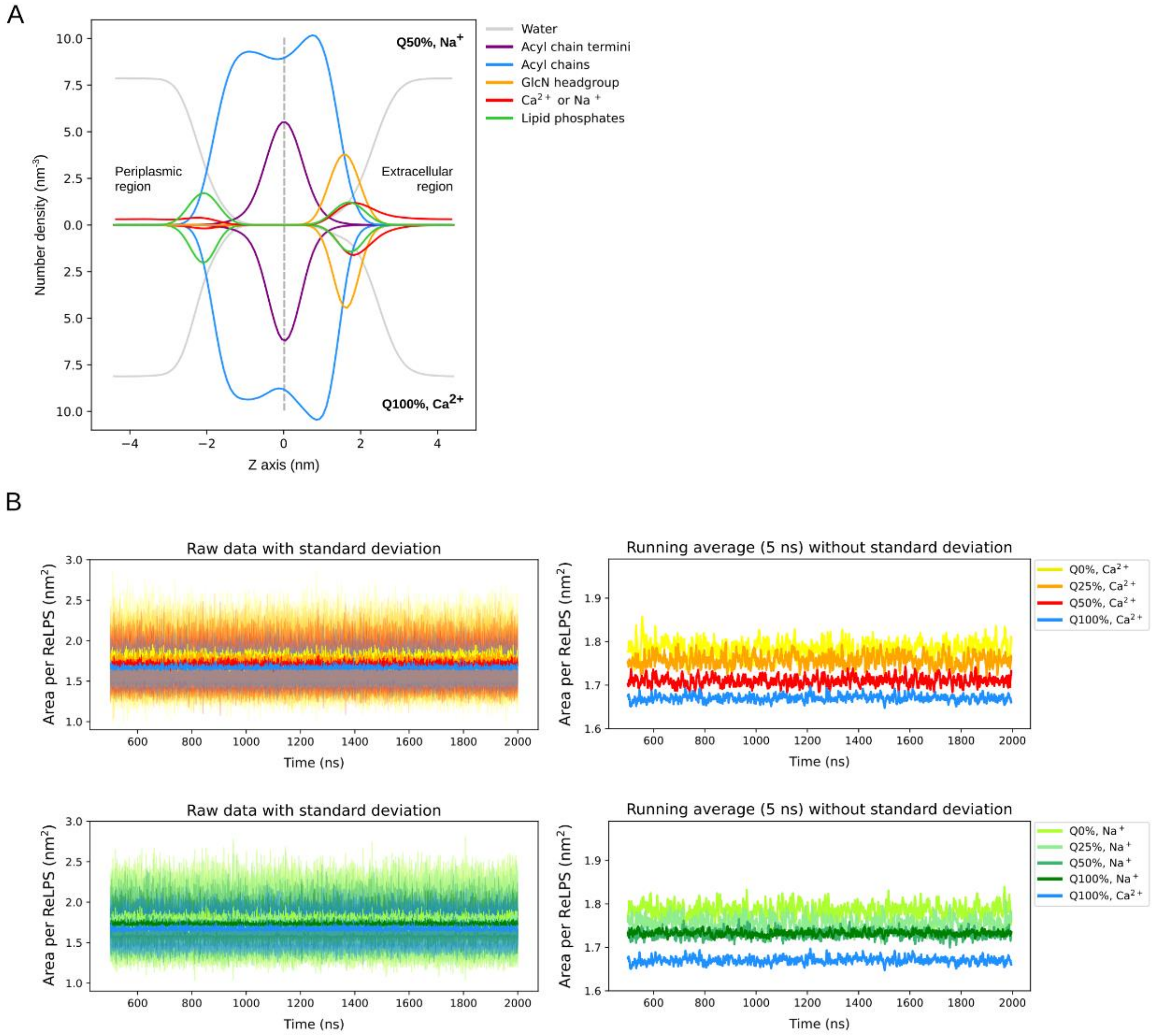

S8. Evaluating the influence of only scaling down charges on Martini 2 ReLPS beads and cations on its partial density profiles and area per lipid. (A) Partial density profiles of various components of the 'Q50%, Na^{+}' system (top half) compared to the (unmodified) reference system 'Q100%, Ca^{2+}' (bottom half). The density profiles are such that the hydrophobic core of the membrane is located at the i.e. Z distance = 0 nm (grey dotted line), and the periplasmic and extracellular regions are located on the left and right hand side of the dotted line respectively. **(B)** APL versus time averaged across simulation replicas of the various systems. The reference system 'Q100%, Ca^{2+}' is shown in blue. For visual clarity, in addition to the APL with standard deviation (left), we show the running average (over 5 ns windows) without standard deviation (right). The plots are further split into two showing APL data from simulations with Ca^{2+} and Na^{+} neutralizing cations.

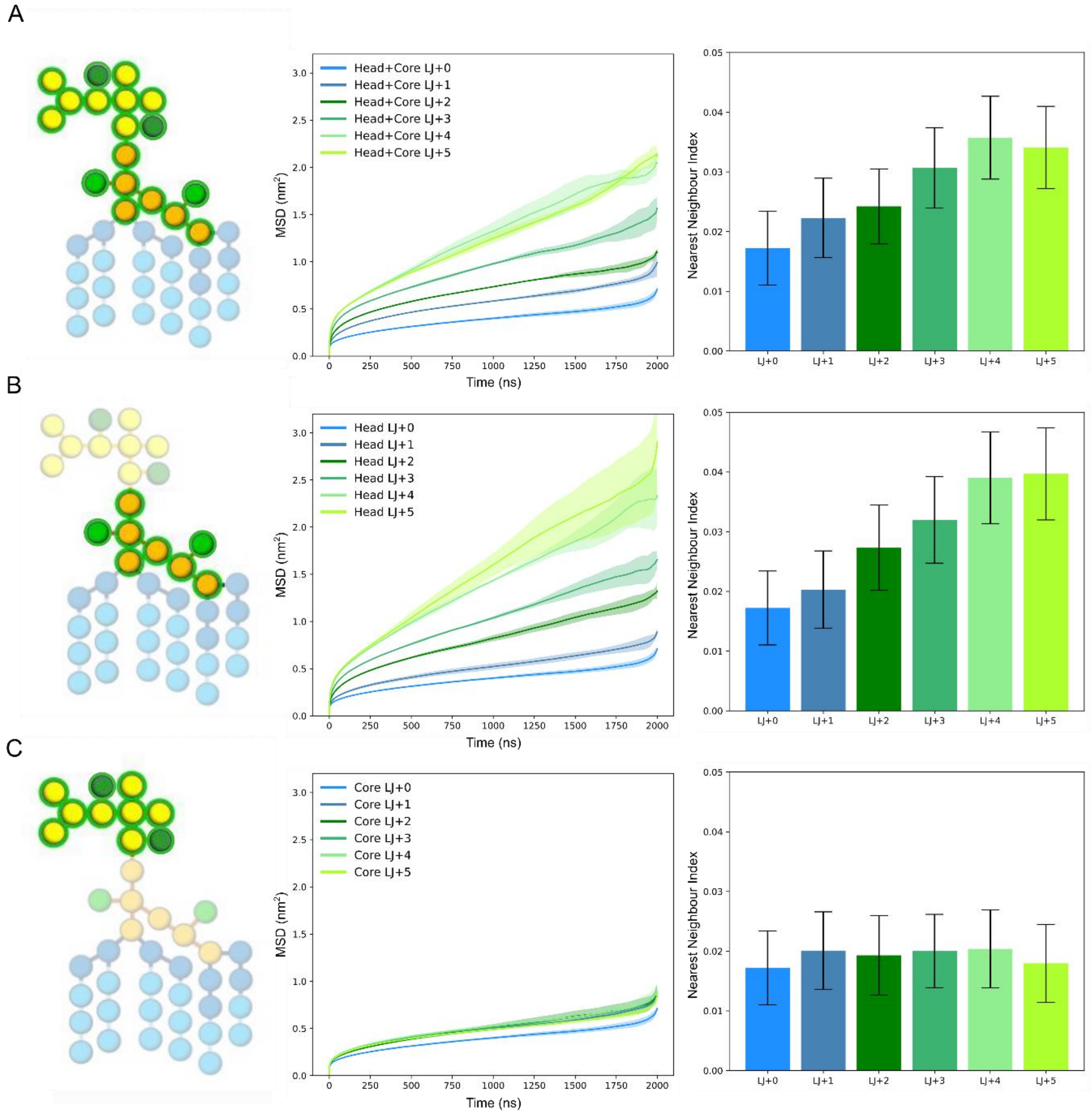

S9. Evaluating the influence of only scaling down LJ potentials on Martini 2 ReLPS beads on its MSD and NNI. MSD and NNI data are shown in the middle and on the right respectively for each combination of LJ-modified beads (scaled down up to 5 levels) in the Martini 2 ReLPS molecule i.e. **(A)** the GlcN headgroup and the Kdo core, **(B)** only the GlcN headgroup, **(C)** only the Kdo core. The LJ-modified beads are highlighted and shown within a green circle in the schematic diagrams on the left.

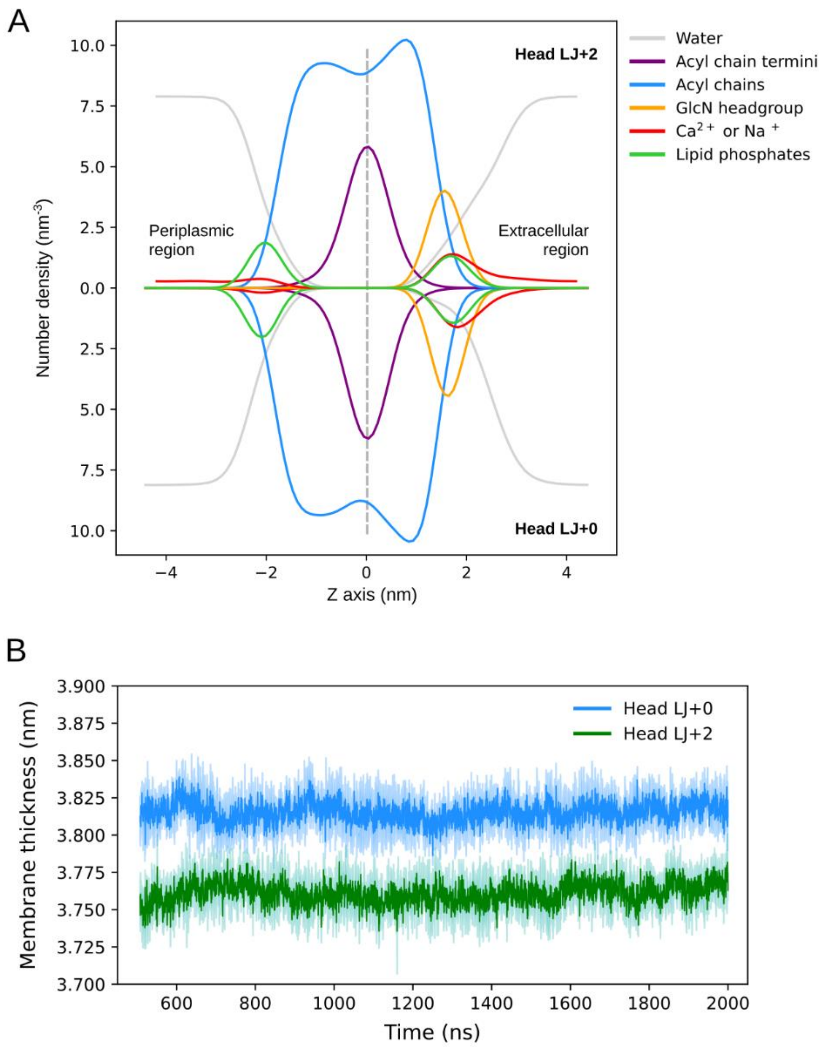

S10. Evaluating the influence of scaling down modifying LJ potentials on Martini 2 ReLPS beads on its partial density profiles and membrane thickness. (A) Partial density profiles of various components of the 'Head LJ+2' system (top half) compared to those of the (unmodified) reference system 'Q100%, Ca^{2+}' (bottom half). The density profiles are centered such that the hydrophobic core of the membrane is located at the center i.e. Z distance = 0 nm (grey dotted line), and the periplasmic and extracellular regions are located on the left and right hand side of the center respectively. **(B)** Membrane thickness versus time (starting from 500 ns) calculated for the 'Head LJ+2' system (dark green) and compared to that of the reference system 'Head LJ+0' (blue).

A

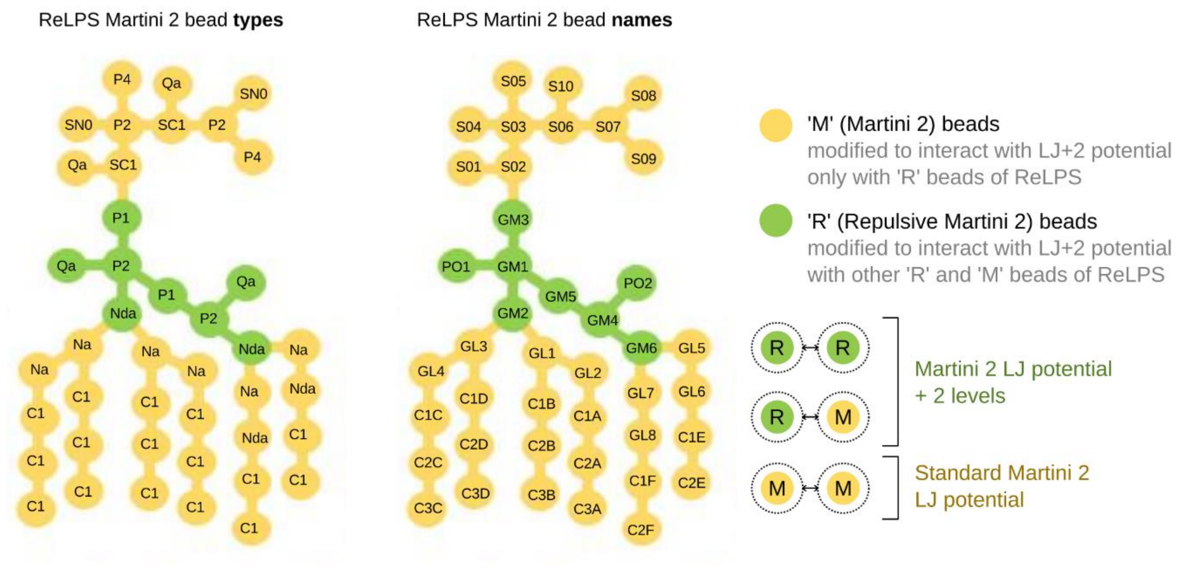

B

Standard Martini 2 ReLPS — **Fast Martini 2 ReLPS**

Standard Martini 2 ReLPS

	Qa	Nda	P1	P2
**Qa**	1.0	1.0	1.0	1.0
**Nda**	1.0	2.0	2.0	2.0
**P1**	1.0	2.0	2.0	2.0
**P2**	1.0	2.0	2.0	2.0

R ⟷ R LJ potential level

	RQa	RNda	RP1	RP2
**RQa**	3.0	3.0	3.0	3.0
**RNda**	3.0	4.0	4.0	4.0
**RP1**	3.0	4.0	4.0	4.0
**RP2**	3.0	4.0	4.0	4.0

R ⟷ R Altered ε value

	RQa	RNda	RP1	RP2
**RQa**	0.80	0.80	0.80	0.80
**RNda**	0.80	0.77	0.77	0.77
**RP1**	0.80	0.77	0.77	0.77
**RP2**	0.80	0.77	0.77	0.77

Standard Martini 2 ReLPS

	Qa	Nda	P1	P2
**Qa**	1.0	1.0	1.0	1.0
**Na**	3.0	2.0	2.0	2.0
**Nda**	1.0	2.0	2.0	2.0
**P2**	1.0	2.0	2.0	2.0
**P4**	0.0	3.0	2.0	2.0
**C1**	9.0	6.0	6.0	7.0
**SC1**	9.0	6.0	6.0	7.0
**SN0**	4.0	4.0	3.0	3.0

M ⟷ R LJ potential level

	RQa	RNda	RP1	RP2
**MQa**	3.0	3.0	3.0	3.0
**MNa**	5.0	4.0	4.0	4.0
**MNda**	3.0	4.0	4.0	4.0
**MP2**	3.0	4.0	4.0	4.0
**MP4**	2.0	5.0	4.0	4.0
**MC1**	9.0	8.0	8.0	9.0
**MSC1**	9.0	8.0	8.0	9.0
**MSN0**	6.0	6.0	5.0	5.0

M ⟷ R Altered ε value

	RQa	RNda	RP1	RP2
**MQa**	0.80	0.80	0.80	0.80
**MNa**	0.77	0.77	0.77	0.77
**MNda**	0.80	0.77	0.77	0.77
**MP2**	0.80	0.77	0.77	0.77
**MP4**	0.80	0.77	0.77	0.77
**MC1**	1.00	0.74	0.74	0.87
**MSC1**	1.00	0.74	0.74	0.87
**MSN0**	0.77	0.77	0.77	0.77

LJ potential levels

Level	Description
0.0	supra attractive
0.5	
1.0	attractive
1.5	
2.0	almost attractive
2.5	
3.0	semi attractive
3.5	
4.0	intermediate
4.5	
5.0	almost intermediate
5.5	
6.0	semi repulsive
6.5	
7.0	almost repulsive
7.5	
8.0	repulsive
8.5	
9.0	super repulsive

S11. Schematic illustrations showing the modified Martini 2 bead names and types, and the Lennard-Jones (LJ) interaction matrix compared between the standard and fast Martini 2 ReLPS parameters. (A) ReLPS labelled as per the standard Martini 2 bead names and types. These beads are coloured according to our parametrization of the fast Martini 2 ReLPS. The headgroup beads (green) are named with a prefix ‘R’ given that they undergo a repulsion of 2 levels compared to standard Martini 2, and all other beads are named ‘M’ given that they had to be modified considering their potential interactions with the ‘R’ beads. **(B)** Comparison of LJ interaction matrices between the standard and fast Martini 2 ReLPS beads. LJ levels 0 to 9 are coloured on a blue-white-red (low-high) scale. The epsilon values of LJ potentials that are altered by ‘n’ times in the fast ReLPS model with reference to the standard model are also shown in a matrix.

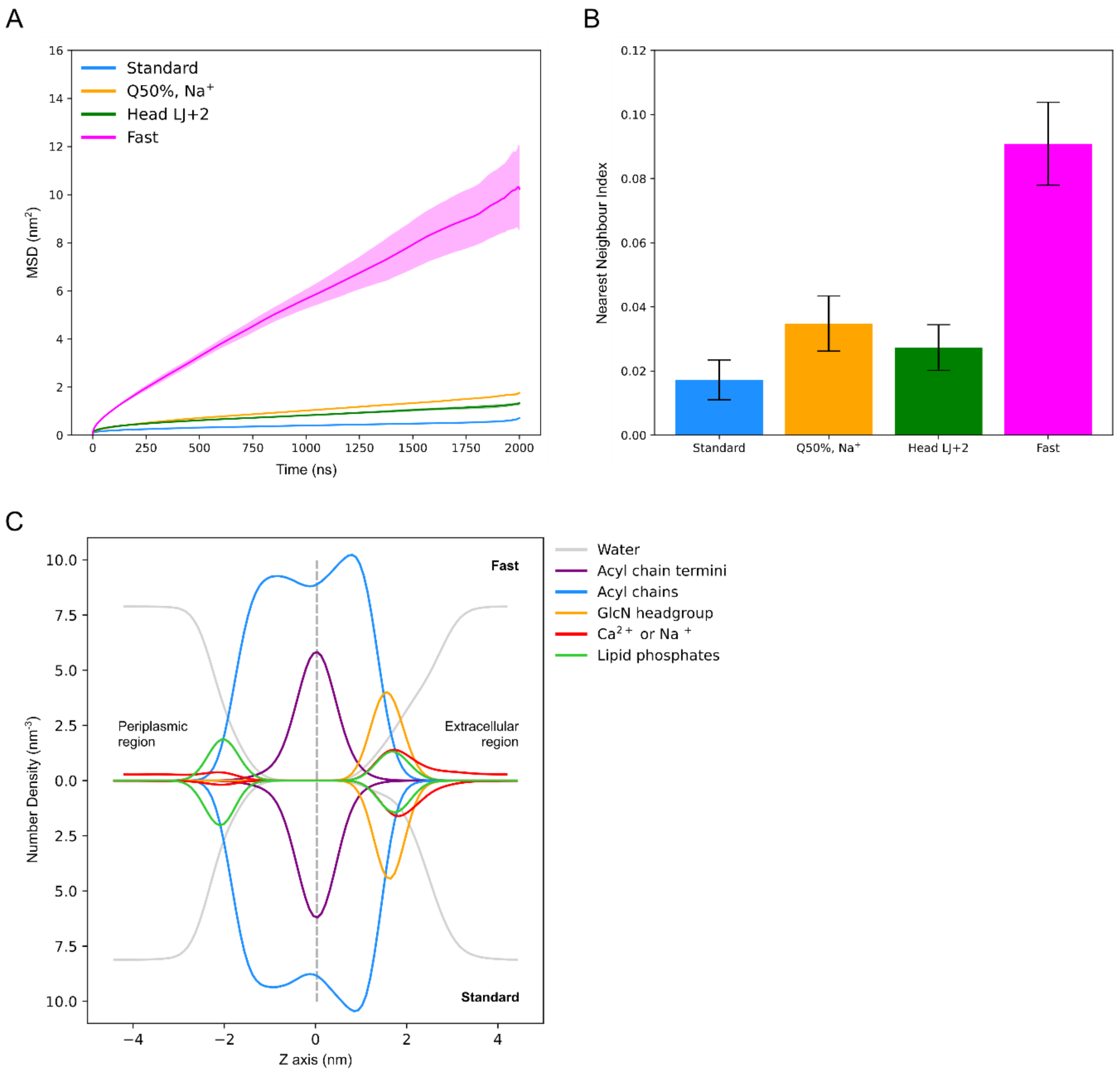

S12. Evaluating the influence of the fast Martini 2 ReLPS parameters on its MSD, NNI, and partial densities. (A) Comparing the MSDs and **(B)** NNI of (1) fast Martini 2 ReLPS (magenta), (2) ReLPS with only their charges modified i.e. 'Q50% Na^{+}' (orange), (3) ReLPS with only their LJ modified i.e. 'Head LJ+2' (green), and (4) the reference system with standard Martini 2 parameters (blue). **(C)** Partial density profiles of various components of the system containing fast Martini 2 ReLPS (top half of the plot) compared to those of the standard Martini 2 (unmodified) system (bottom half of the plot). The density profiles are such that the hydrophobic core of the membrane is located at the i.e. Z distance = 0 nm (grey dotted line), and the periplasmic and extracellular regions are located on the left and right hand side of the centre respectively.

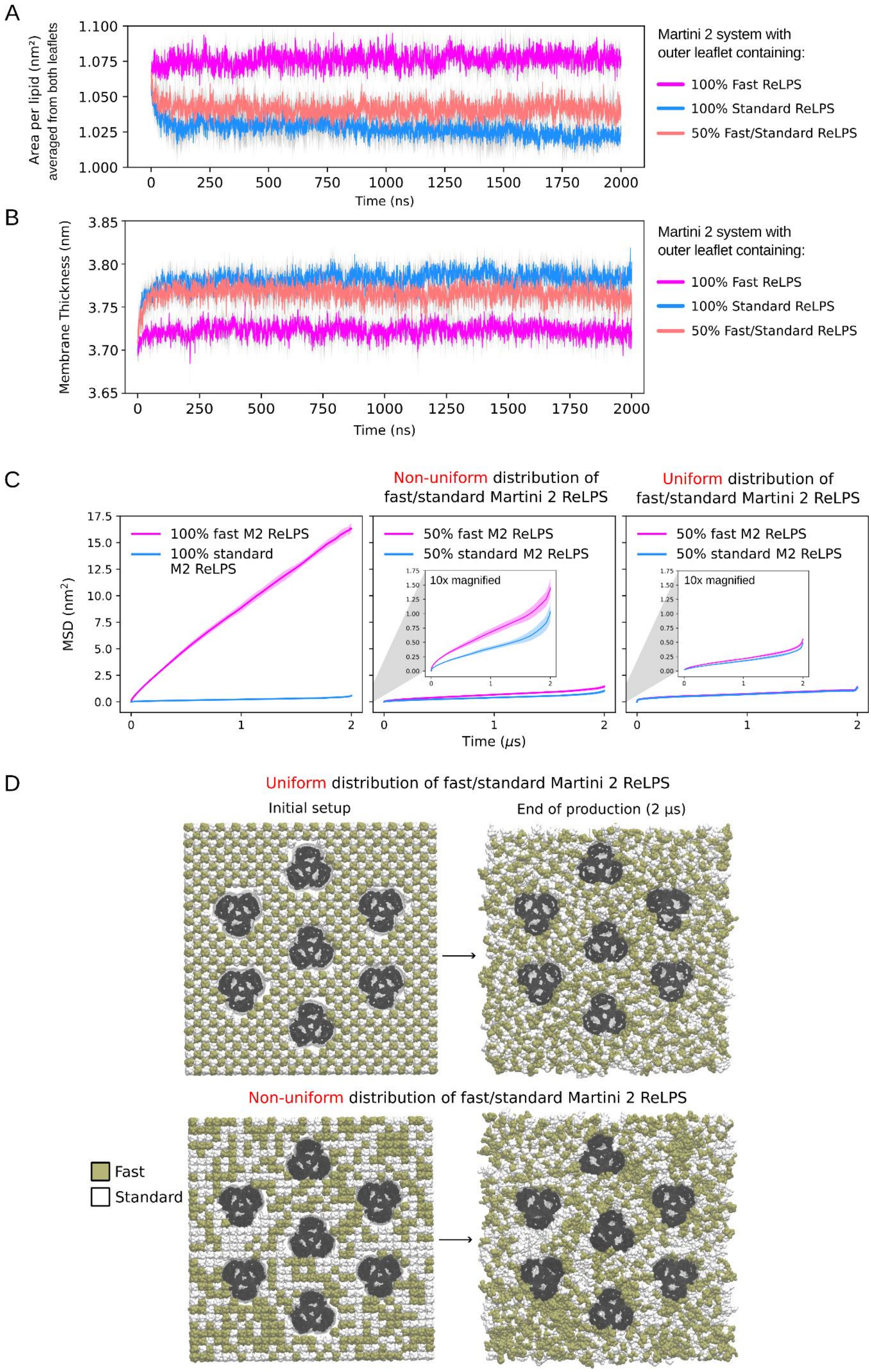
A
Area per lipid (nm²) averaged from both leaflets
Time (ns)
Martini 2 system with outer leaflet containing:
100% Fast ReLPS
100% Standard ReLPS
50% Fast/Standard ReLPS
B
Membrane Thickness (nm)
Time (ns)
Martini 2 system with outer leaflet containing:
100% Fast ReLPS
100% Standard ReLPS
50% Fast/Standard ReLPS
C
Non-uniform distribution of fast/standard Martini 2 ReLPS
Uniform distribution of fast/standard Martini 2 ReLPS
MSD (nm²)
100% fast M2 ReLPS
100% standard M2 ReLPS
50% fast M2 ReLPS
50% standard M2 ReLPS
10x magnified
Time (μs)
D
Uniform distribution of fast/standard Martini 2 ReLPS
Initial setup
End of production (2 μs)
Non-uniform distribution of fast/standard Martini 2 ReLPS
Fast
Standard

S13. Comparing APL, membrane thickness, and MSDs of Martini 2 systems containing OMPs surrounded by 100% fast ReLPS, 100% standard ReLPS, and 50% fast/standard ReLPS in the outer leaflet. (A) Averaged APL from both membrane leaflets and **(B)** membrane thickness versus time of systems containing 100% fast ReLPS (magenta), 100% standard ReLPS (cyan), and 50% fast/standard ReLPS (light red) in the outer leaflet of the outer membrane model. Standard deviations for each data line is shown in light grey. **(C)** MSDs versus time compared between 100% fast and 100% standard Martini 2 (left). In the 50% fast/standard ReLPS-containing systems, two initial setups were obtained i.e. a non-uniform and a uniform distribution of Martini 2 fast/standard ReLPS. From each of these simulations, MSDs were calculated separately for the fast ReLPS molecules and for the standard ReLPS molecules. A comparison of MSDs between the fast and standard ReLPS molecules is made from the simulations starting with the non-uniform distribution (center) and the uniform distribution (right). **(D)** Snapshots of the initial setup (left) and final frame (right) of simulations starting with a uniform distribution (top) and non-uniform distribution (bottom) of fast and standard ReLPS molecules.

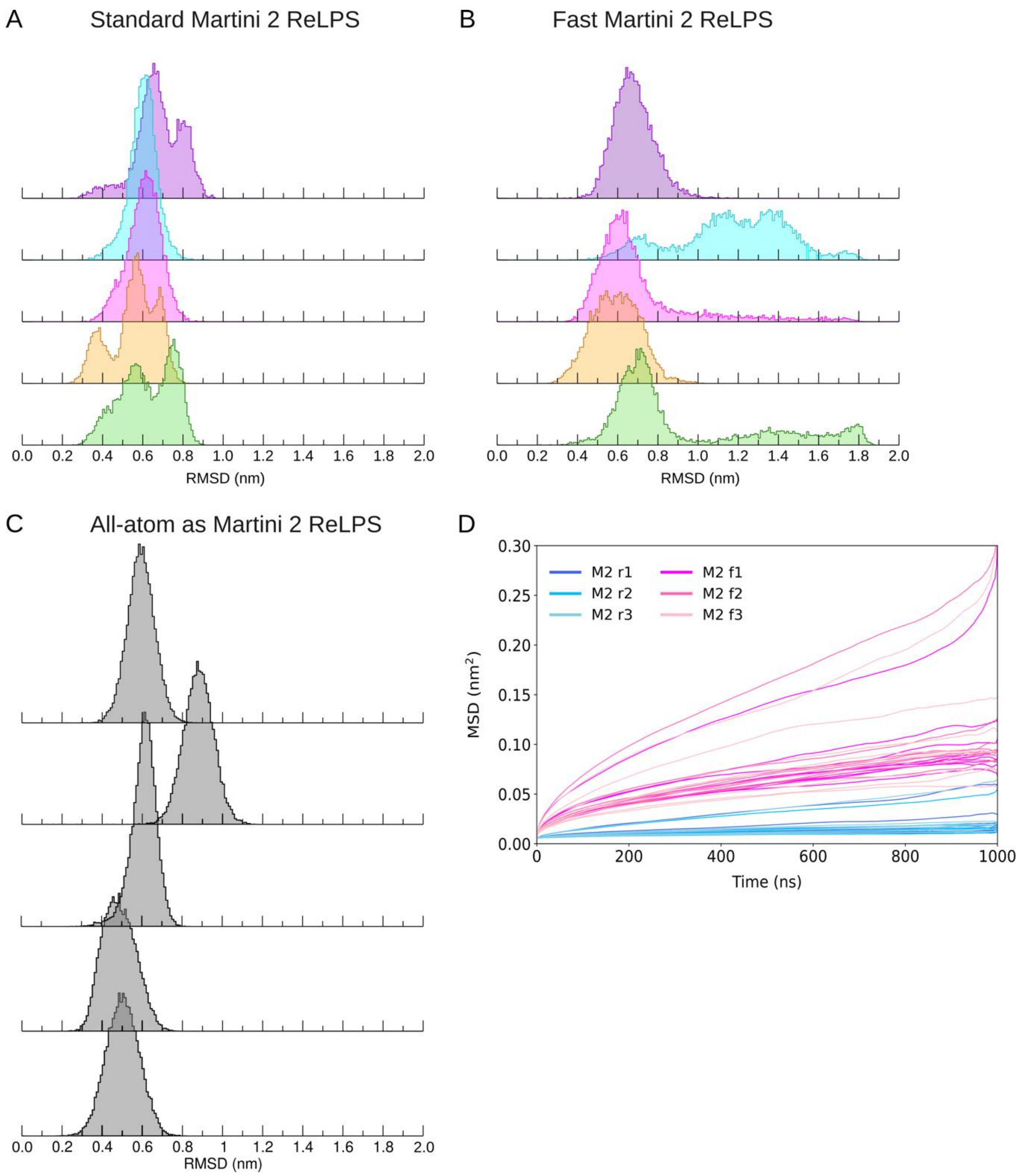

S14. Internal flexibility and lateral mobility of ReLPS in a 100% ReLPS symmetric bilayer. (A,B,C) RMSD histograms for 5 individual ReLPS molecules. The full 10 μs trajectory was analyzed for the **(A)** standard Martini 2 and **(B)** fast Martini 2 models. RMSD was calculated on the whole molecule after fitting in the central region (GlcN and phosphates). The atomistic simulation was mapped into Martini 2 where 5 randomly chosen individual ReLPS molecules were analyzed as explained and shown in **(C)**. **(D)** Lateral MSD of ReLPS in replicas of standard (M2 r1-3) and fast Martini 2 (M2 f1-3) ReLPS computed independently for 10 non-overlapping 1 μs-long trajectory segments.

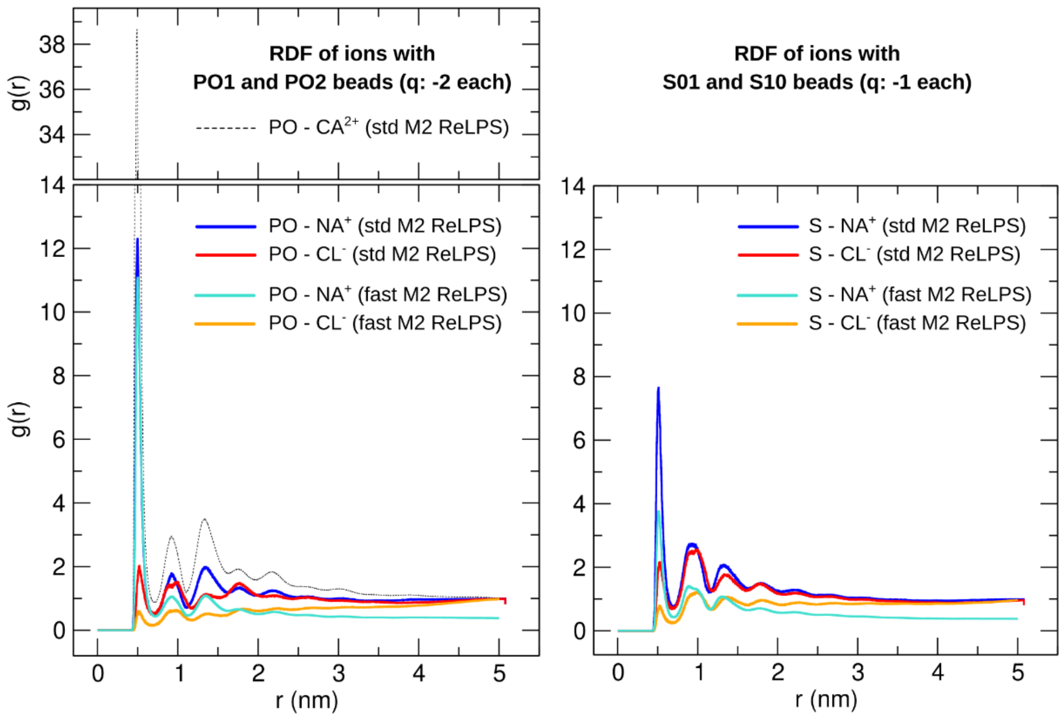

S15. Comparing ReLPS phosphate-ion interactions between standard and fast Martini 2 models. RDFs of (1) divalent, monovalent cations, and monovalent anions with ReLPS phosphate beads (left), and (2) monovalent cations and anions with charged glycan (S) beads in the ReLPS core.

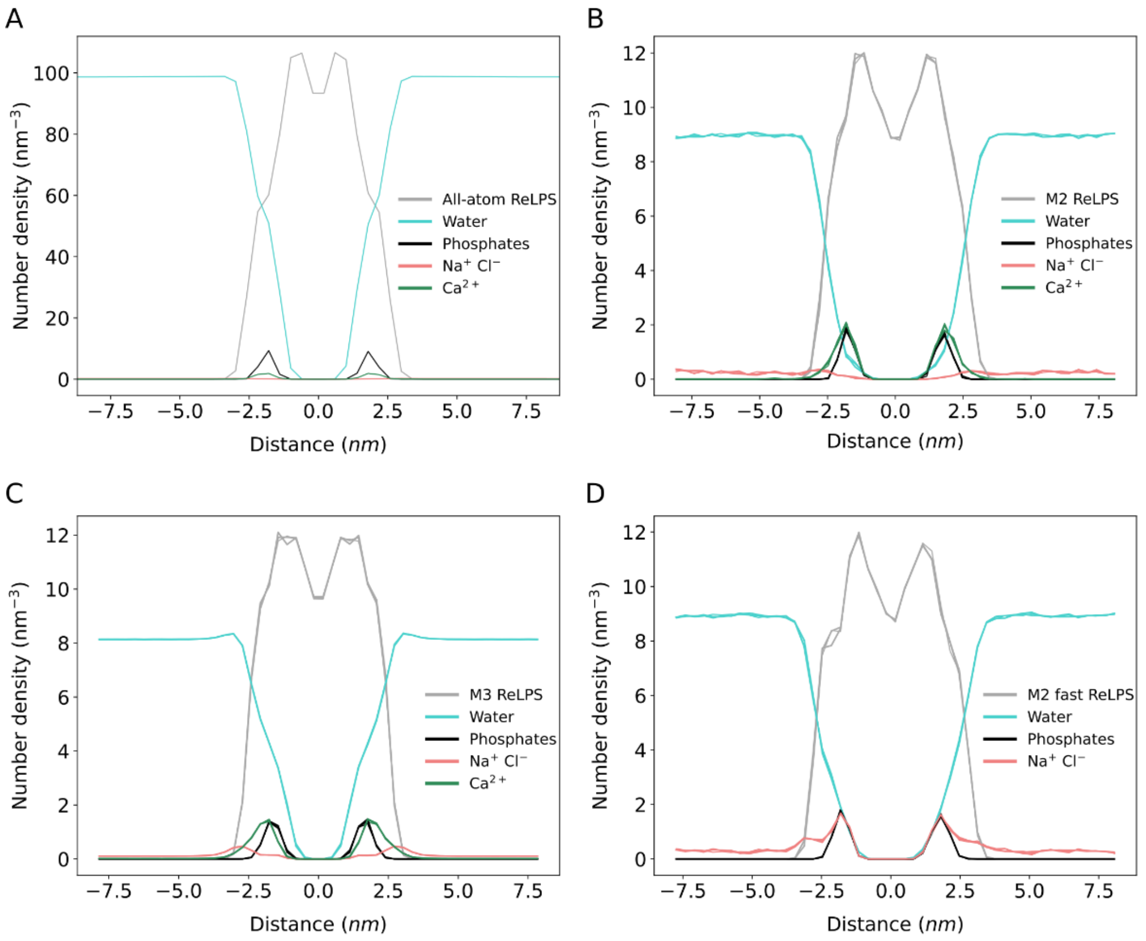

S16. Lateral molecular organization of a 100% ReLPS symmetric bilayer. Lateral number densities of different molecular groups of interest along the membrane normal, computed for **(A)** the all-atom simulation, **(B)** standard Martini 2**, (C)** Martini 3, and **(D)** fast Martini 2. Distance = 0 nm corresponds to the centre of the bilayer along the Z axis.

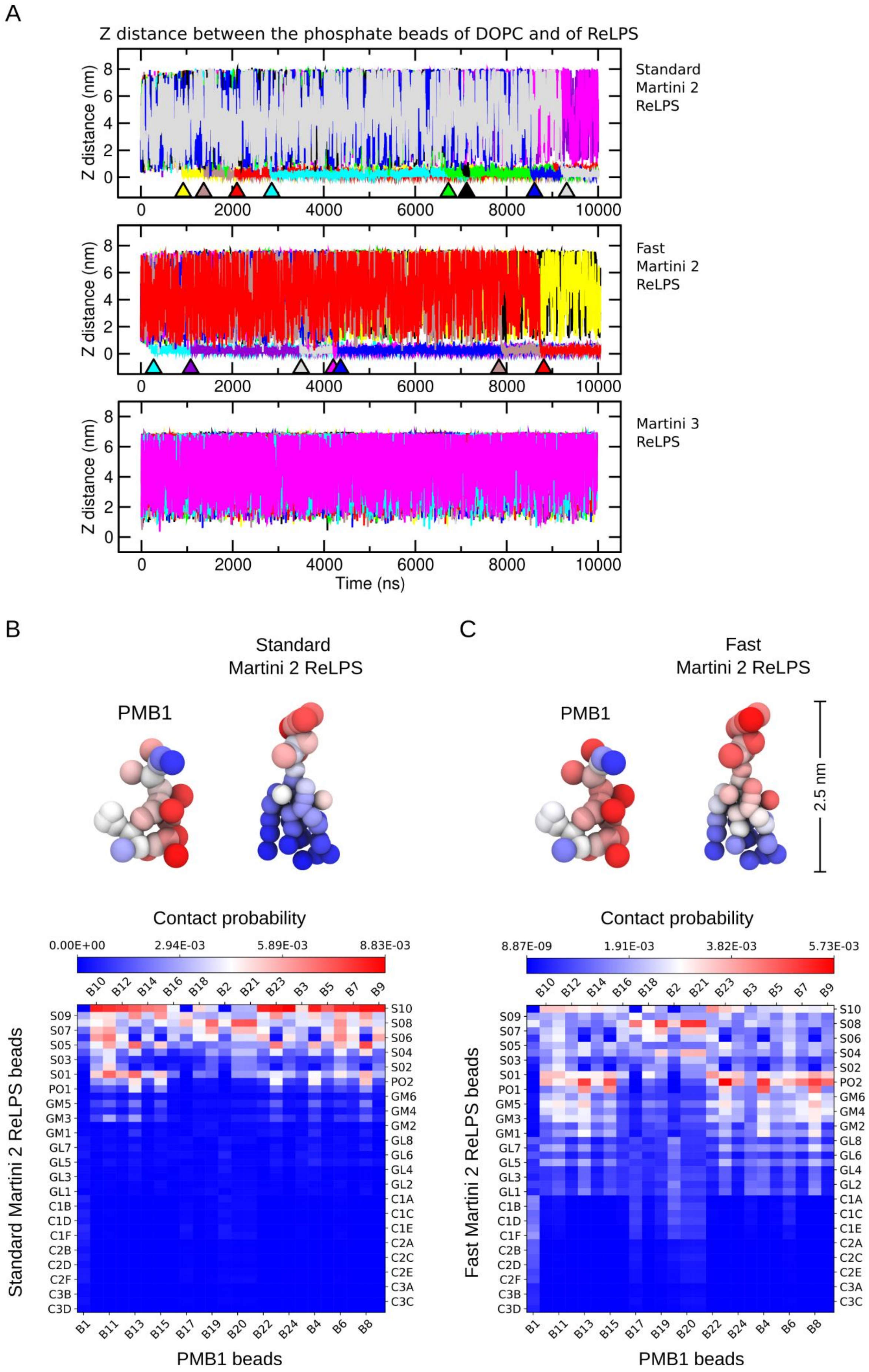

A
Z distance between the phosphate beads of DOPC and of ReLPS
Standard Martini 2 ReLPS
Fast Martini 2 ReLPS
Martini 3 ReLPS
Z distance (nm)
Time (ns)
B
Standard Martini 2 ReLPS
PMB1
C
Fast Martini 2 ReLPS
2.5 nm
Contact probability
0.00E+00
2.94E-03
5.89E-03
8.83E-03
8.87E-09
1.91E-03
3.82E-03
5.73E-03
Standard Martini 2 ReLPS beads
Fast Martini 2 ReLPS beads
PMB1 beads

S17. DOPC and PMB1 insertion tests conducted with standard and fast Martini 2 ReLPS. (A) Distance along the Z dimension (perpendicular to the membrane) versus time calculated between the phosphate bead(s) of the (single) DOPC molecule and of the ReLPS molecules in the outer leaflet of the membrane. **(B)** Average number of contacts of PMB1 beads with standard Martini 2 ReLPS beads are mapped onto their respective coarse-grained structures on a blue-white-red (low-high) scale. **(C)** The same is performed for contacts between PMB1 and fast Martini 2 ReLPS. For both (A) and (B), their respective contact probabilities are plotted on a matrix and shown below using the same colour scale. For visual clarity, labels are alternated between opposite axes.

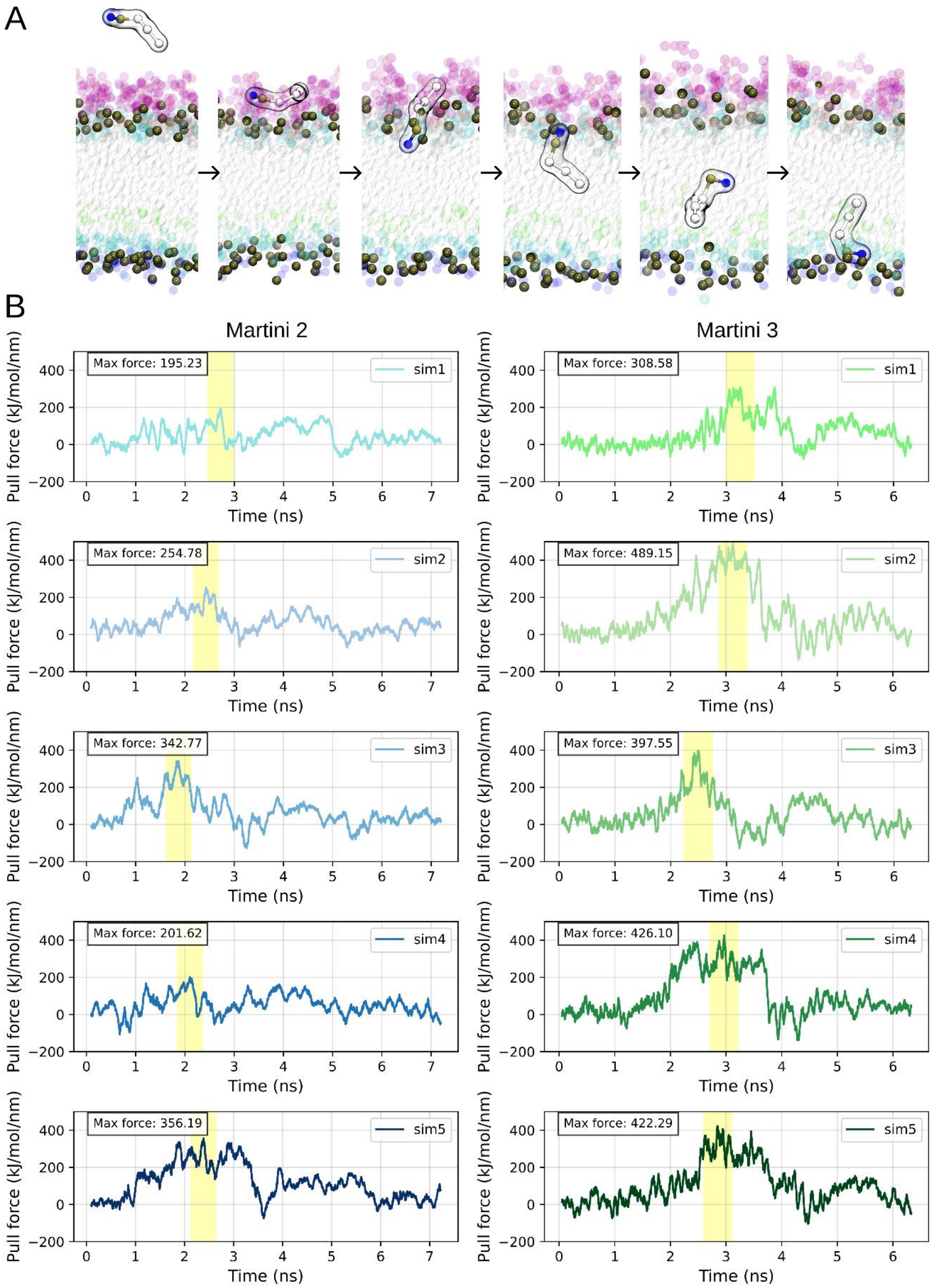

S18. Steered MD simulations of DPC. (A) Snapshots taken from steered MD simulations of DPC in Martini 3. **(B)** Data showing the change in pulling force versus time while DPC was steered from water into the membrane using Martini 2 (left) and Martini 3 ReLPS (right) models. In each plot, the maximum force experienced by DPC is labelled and corresponds to the yellow region in each plot.

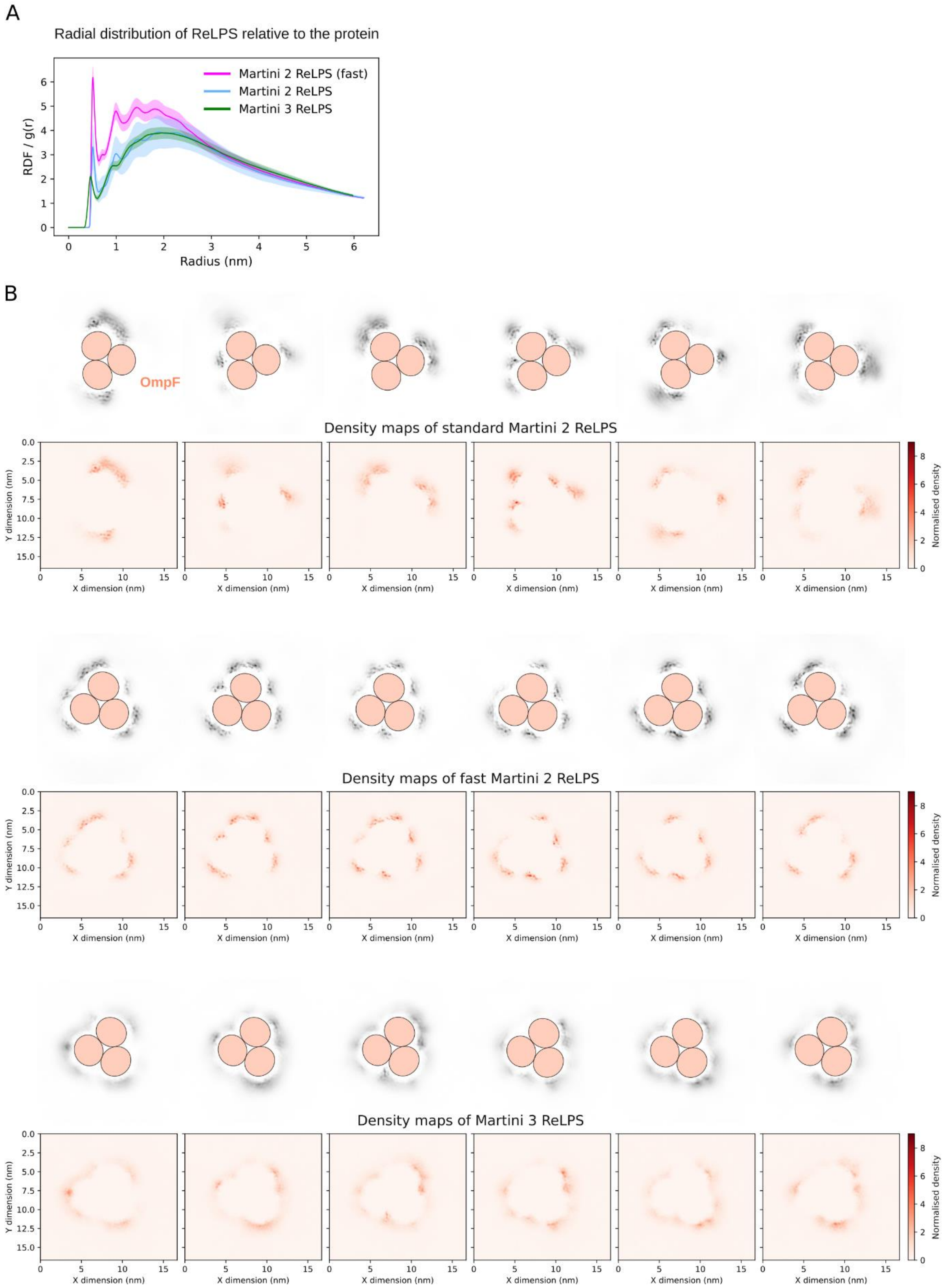

S19. Densities of ReLPS relative to OmpF in an OM model with 2% ReLPS in the outer leaflet. (A) Radial distribution functions and (B) 2D density maps of the Martini 2 and 3 ReLPS models with reference to an OmpF trimer.

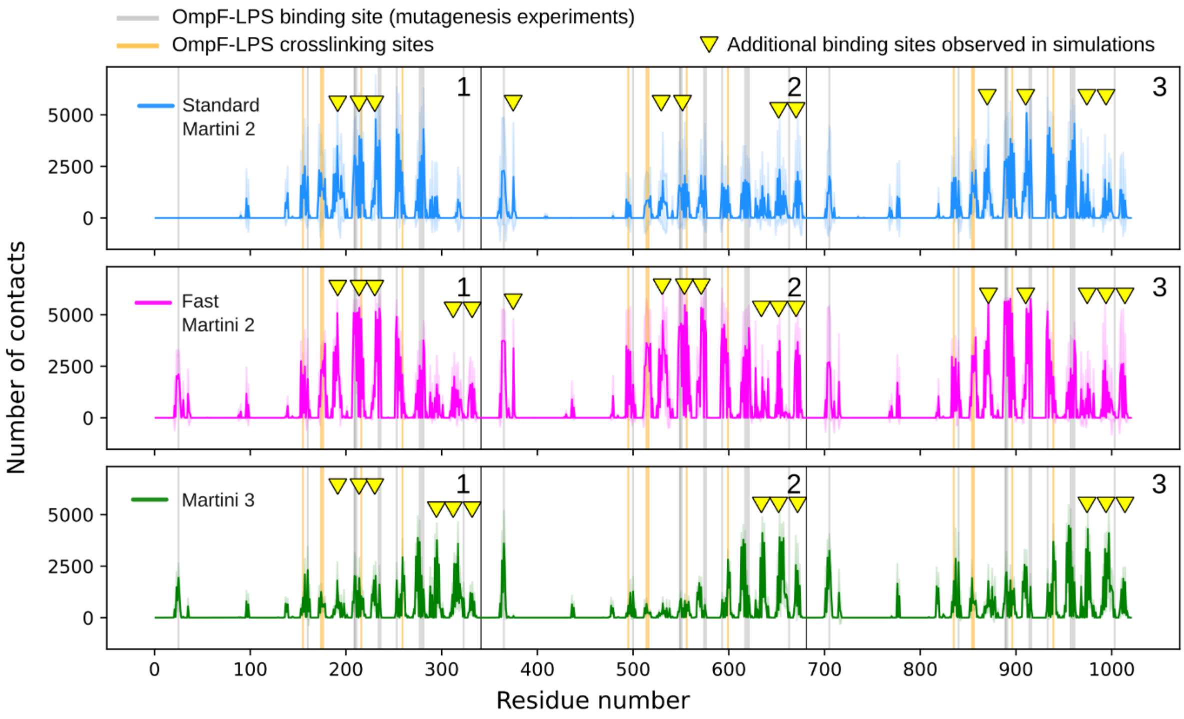

S20. Number of OmpF-ReLPS contacts in standard Martini 2, fast Martini 2, and Martini 3 simulations with 2% ReLPS concentration in the outer leaflet. For standard Martini 2 (upper panel), fast Martini 2 (middle), and Martini 3 (bottom), the contacts are shown in blue, magenta, and green respectively. Black lines are shown to differentiate between the residues of OmpF monomers. Grey lines indicate the residues that are proposed to contribute to LPS by mutagenesis experiments [14]. Orange lines indicate the residues that are involved in LPS binding as shown by OmpF-LPS crosslinking experiments [15]. The yellow triangles indicate newly identified OmpF residues from simulations that show a large number of contacts with ReLPS in addition to the residues that align with experimental data.

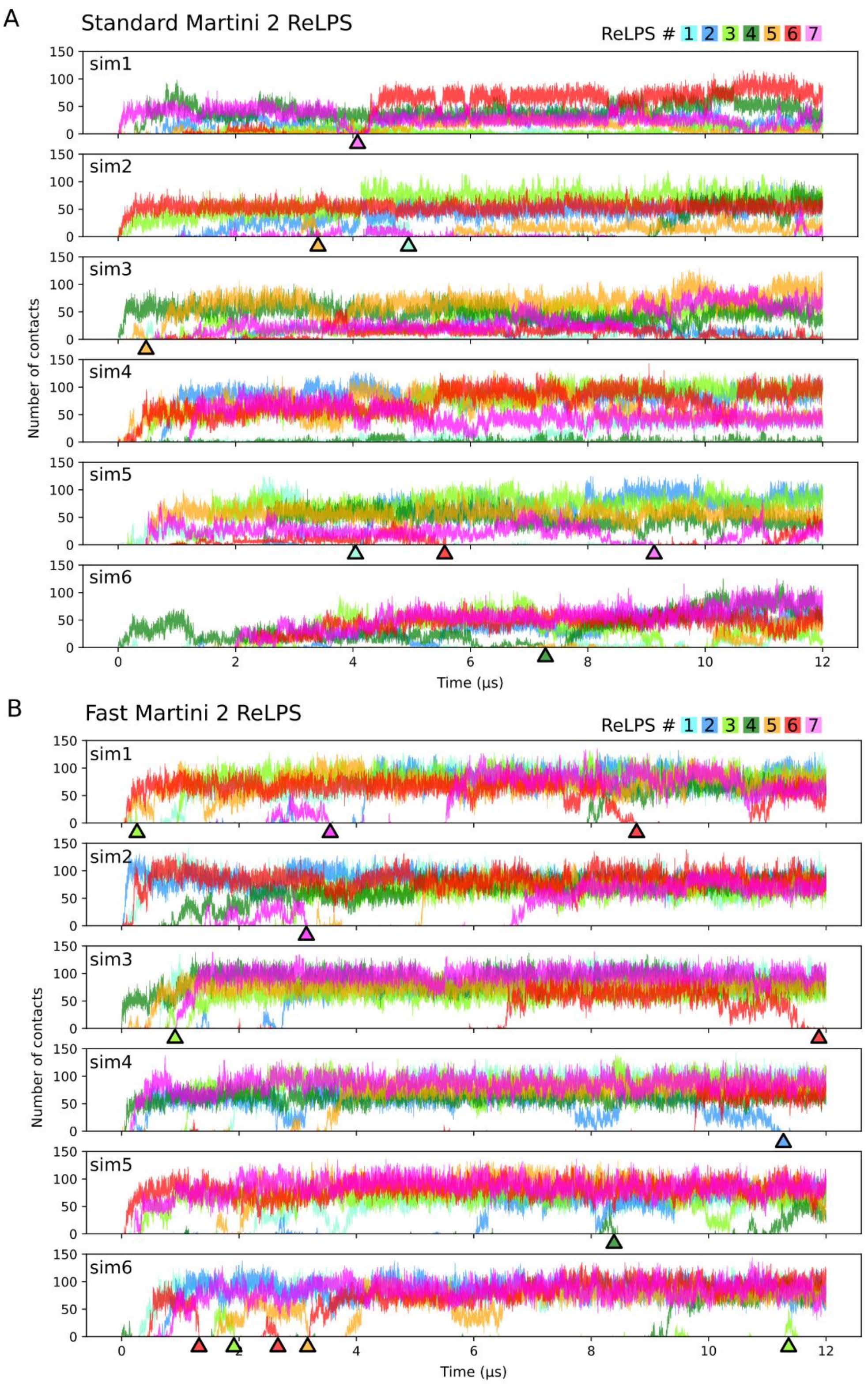

S21. Dissociation events between Martini 2 ReLPS models and OmpF in an OM model with 2% ReLPS in the outer leaflet. Number of OmpF-ReLPS contacts versus time shown from three individual simulations conducted with **(A)** standard and **(B)** fast Martini 2 ReLPS. Some dissociation events are indicated by arrows below the respective plots with their colours matching with that of the ReLPS molecule number.

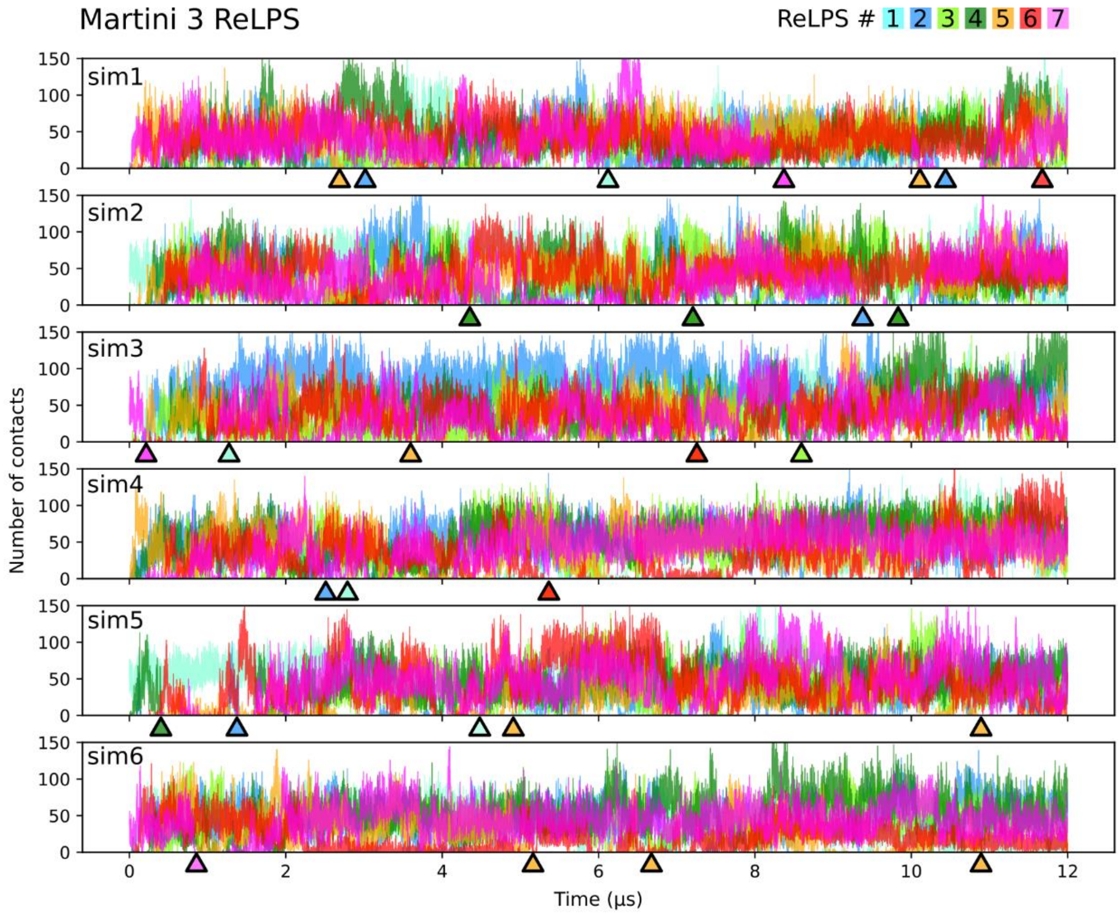

S22. Dissociation events between our Martini 3 ReLPS model and OmpF in an OM model with 2% ReLPS in the outer leaflet. Number of OmpF-ReLPS contacts versus time shown from three individual simulations conducted with our Martini 3 ReLPS model. Some dissociation events are indicated by arrows below the respective plots with their colours matching with that of the ReLPS molecule number.

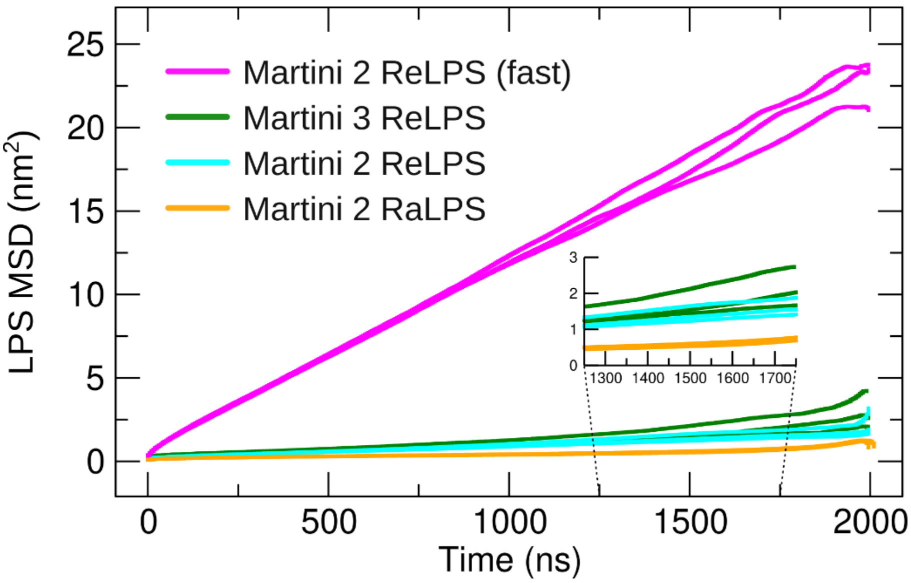

S23. Comparing the lateral diffusion of the standard Martini 2 ReLPS and RaLPS, fast Martini 2 ReLPS, our Martini 3 ReLPS models. This was done by computing their MSD versus time from the OMP island simulations. The lateral MSDs computed from each simulation replica are plotted individually.

4. Supplementary References

(1) Nosé, S. A Unified Formulation of the Constant Temperature Molecular Dynamics Methods. *J Chem Phys* **1984**, *81* (1), 511–519. https://doi.org/10.1063/1.447334.

(2) Hoover, W. G. Canonical Dynamics: Equilibrium Phase-Space Distributions. *Phys Rev A (Coll Park)* **1985**, *31* (3), 1695. https://doi.org/10.1103/PhysRevA.31.1695.

(3) Parrinello, M.; Rahman, A. Polymorphic Transitions in Single Crystals: A New Molecular Dynamics Method. *J Appl Phys* **1981**, *52* (12), 7182–7190. https://doi.org/10.1063/1.328693.

(4) Hess, B.; Bekker, H.; Berendsen, H. J. C.; Fraaije, J. G. E. M. LINCS: A Linear Constraint Solver for Molecular Simulations. *J Comput Chem* **1998**, *18* (12), 1463–1472. https://doi.org/https://doi.org/10.1002/(SICI)1096-987X(199709)18:12<1463::AID-JCC4>3.0.CO;2-H.

(5) Darden, T.; York, D.; Pedersen, L. Particle Mesh Ewald: An N·log(N) Method for Ewald Sums in Large Systems. *J Chem Phys* **1993**, *98* (12), 10089–10092. https://doi.org/10.1063/1.464397.

(6) Berendsen, H. J. C.; Postma, J. P. M.; Van Gunsteren, W. F.; Dinola, A.; Haak, J. R. Molecular Dynamics with Coupling to an External Bath. *J Chem Phys* **1984**, *81* (8), 3684–3690. https://doi.org/10.1063/1.448118.

(7) Bussi, G.; Donadio, D.; Parrinello, M. Canonical Sampling through Velocity Rescaling. *Journal of Chemical Physics* **2007**, *126* (1). https://doi.org/10.1063/1.2408420/186581.

(8) Abraham, M. J.; Murtola, T.; Schulz, R.; Páll, S.; Smith, J. C.; Hess, B.; Lindah, E. GROMACS: High Performance Molecular Simulations through Multi-Level Parallelism from Laptops to Supercomputers. *SoftwareX* **2015**, *1–2*, 19–25. https://doi.org/10.1016/J.SOFTX.2015.06.001.

(9) Song, W.; Corey, R. A.; Ansell, T. B.; Cassidy, C. K.; Horrell, M. R.; Duncan, A. L.; Stansfeld, P. J.; Sansom, M. S. P. PyLipID: A Python Package for Analysis of Protein-Lipid Interactions from Molecular Dynamics Simulations. *J Chem Theory Comput* **2022**, *18* (2), 1188–1201. https://doi.org/10.1021/ACS.JCTC.1C00708/.

(10) Buchoux, S. FATSLiM: A Fast and Robust Software to Analyze MD Simulations of Membranes. *Bioinformatics* **2017**, *33* (1), 133–134. https://doi.org/10.1093/BIOINFORMATICS/BTW563.

(11) Humphrey, W.; Dalke, A.; Schulten, K. VMD: Visual Molecular Dynamics. *J Mol Graph* **1996**, *14* (1), 33–38. https://doi.org/10.1016/0263-7855(96)00018-5.

(12) Harris, C. R.; Millman, K. J.; van der Walt, S. J.; Gommers, R.; Virtanen, P.; Cournapeau, D.; Wieser, E.; Taylor, J.; Berg, S.; Smith, N. J.; Kern, R.; Picus, M.; Hoyer, S.; van Kerkwijk, M. H.; Brett, M.; Haldane, A.; del Río, J. F.; Wiebe, M.; Peterson, P.; Gérard-Marchant, P.; Sheppard, K.; Reddy, T.; Weckesser, W.; Abbasi, H.; Gohlke, C.; Oliphant, T. E. Array Programming with NumPy. *Nature* **2020**, *585* (7825), 357–362. https://doi.org/10.1038/s41586-020-2649-2.

(13) Hunter, J. D. Matplotlib: A 2D Graphics Environment. *Comput Sci Eng* **2007**, *9* (3), 90–95. https://doi.org/10.1109/MCSE.2007.55.

(14) Shearer, J.; Marzinek, J. K.; Bond, P. J.; Khalid, S. Molecular Dynamics Simulations of Bacterial Outer Membrane Lipid Extraction: Adequate Sampling? *J Chem Phys* **2020**, *153* (4). https://doi.org/10.1063/5.0017734.
